# Highly Adaptable Triple-Negative Breast Cancer Cells as a Functional Model for Testing Anticancer Agents

**DOI:** 10.1371/journal.pone.0109487

**Published:** 2014-10-03

**Authors:** Balraj Singh, Anna Shamsnia, Milan R. Raythatha, Ryan D. Milligan, Amanda M. Cady, Simran Madan, Anthony Lucci

**Affiliations:** Department of Surgical Oncology, and Morgan Welch Inflammatory Breast Cancer Research Program and Clinic, The University of Texas MD Anderson Cancer Center, Houston, Texas, United States of America; Roswell Park Cancer Institute, United States of America

## Abstract

A major obstacle in developing effective therapies against solid tumors stems from an inability to adequately model the rare subpopulation of panresistant cancer cells that may often drive the disease. We describe a strategy for optimally modeling highly abnormal and highly adaptable human triple-negative breast cancer cells, and evaluating therapies for their ability to eradicate such cells. To overcome the shortcomings often associated with cell culture models, we incorporated several features in our model including a selection of highly adaptable cancer cells based on their ability to survive a metabolic challenge. We have previously shown that metabolically adaptable cancer cells efficiently metastasize to multiple organs in nude mice. Here we show that the cancer cells modeled in our system feature an embryo-like gene expression and amplification of the fat mass and obesity associated gene *FTO*. We also provide evidence of upregulation of *ZEB1* and downregulation of *GRHL2* indicating increased epithelial to mesenchymal transition in metabolically adaptable cancer cells. Our results obtained with a variety of anticancer agents support the validity of the model of realistic panresistance and suggest that it could be used for developing anticancer agents that would overcome panresistance.

## Introduction

Our understanding of cancer has advanced tremendously over the last four decades. However, translation of this knowledge into clinical applications to improve treatment outcomes has been slow, particularly for solid tumors. The difficulty stems in large part from the fact that only rare cancer cells (often representing as little as 0.001% of the total cell population) truly drive the disease, particularly metastasis [1]–[4]. These rare special cells are akin to Olympic decathlon winners; such cells may also be the cause of panresistance (resistance to all existing therapies), often seen in patients with advanced disease [5]. The difficulties in overcoming panresistance are best understood in the context of the mechanisms of tumor heterogeneity. Previous attempts to address the tumor heterogeneity problem by isolating important subpopulations of cancer cells using a variety of methods achieved various degrees of success. These methods include 1) *in vitro* selection based on the ability of cancer cells to invade the basement membrane, 2) *in vitro* selection based on the ability of cancer cells to grow in soft or hard agar, 3) selection of cancer cells based on their ability to colonize and grow at metastasis sites in nude mice, and 4) more recently, enrichment of cancer stem cells on the basis of specific cell surface markers [6]–[8]. Here, we describe a new strategy for delving deeper into the roots of cancer. Our strategy is based on the hypothesis that decathlon winner cancer cells/roots can resist severe metabolic challenges and this ability can be employed for selecting them. Increasingly, metabolic state is viewed not merely as a recipient of aberrant signaling in cancer but rather as an important driving factor in oncogenesis [e.g., see reference 9]. We applied this knowledge of a linkage between metabolic state and regulatory state to isolate rare cancer cells whose adaptability can drive metastasis.

Since current methods of preclinically evaluating new drug candidates poorly predict treatment response in cancer patients, we are developing a new strategy to test potential therapeutic agents with the goal of better predicting response in patients. The strategy consists of three elements, all aimed at improving the likelihood of accurately determining whether a test therapy can eradicate the roots of a therapy-resistant cancer: 1) selecting a cell line for optimal modeling of mutations and other features that drive therapy resistance in patients, 2) choice of body-like selection strategy to eliminate most cancer cells that would die under nutrient starvation, and 3) equally important, evaluating therapies in long-term assays to accurately predict response in the clinic. Our strategy is focused on modeling cancer roots that are highly abnormal and highly adaptable. The rationale is that if a test therapy is effective against such roots, it is more likely to overcome therapy resistance and succeed in treating cancer patients. Here, we describe the application of this cell-based approach to triple-negative breast cancer (TNBC), which lacks expression of estrogen receptor, expression of progesterone receptor, and HER2 gene amplification. TNBC is an aggressive and heterogeneous breast cancer, with considerable overlap with basal-like breast cancers. The *TP53* tumor suppressor gene is commonly mutated in TNBC [10], [11]. The germ line mutation in the BRCA1 gene is also associated with TNBC. These mutations indicate that TNBC is a disease of genome instability. TNBC-like molecular features have been observed in other cancers, e.g., high-grade serous ovarian carcinoma [11]. We chose to model the roots of TNBC with an intention to contribute to drug discovery efforts against cancers that are very heterogeneous and adaptable.

To model the roots of therapy-resistant TNBC, we chose the SUM149 cell line because it has the following characteristics: 1) origination from a very aggressive human triple-negative inflammatory breast cancer (TN-IBC), 2) several mutations that are often observed in TNBC, including a gain-of-function M237I mutation in the *TP53* gene that may affect protein stability and function similar to the hotspot R175H mutation [12], [13], a defective RB pathway due to p16 deletion, a micro-scale genomic deletion in *PTEN*, 3) *BRCA1* mutation, making the cell line relevant for patients with a defective *BRCA1* gene [14], [15], and 4) one of the highest rates of metastatic ability among TNBC cell lines, as indicated by the ability of the cells to reproducibly metastasize to distant organs from fat pad xenografts in nude mice [16], [17]. It is important to stress that IBC resembles non-inflammatory breast cancers in terms of gene expression patterns; both TNBC and TN-IBC are heterogeneous subgroups [18], [19]. Therefore, the TN-IBC cell line SUM149 is a good model for aggressive, therapy-resistant TNBC. After considering many approaches for isolating the TNBC cells responsible for therapy resistance, we hypothesized that a severe metabolic challenge would select rare cancer cells that can survive a variety of challenges and thus drive metastasis and panresistance. We reasoned that the therapy-resistant cancer cells selected by this approach may be more useful for testing new anticancer agents against the therapy-resistant disease than the resistant cancer cells selected to survive a specific chemotherapeutic or targeted agent. In support of this approach, we recently reported that lack of glutamine (Gln) in culture medium killed more than 99.99% of SUM149 cells. However, the rare cells (0.01%; termed MA for metabolically adaptable) that survive and grow are capable of surviving additional metabolic challenge, i.e., lack of glucose, they are resistant to chemotherapeutic drugs doxorubicin and paclitaxel, and they efficiently metastasize to multiple organs-lungs, liver, brain, and skin-from fat pad xenografts in nude mice [20]. Our results indicated that a robust *in vitro* selection method can be more useful and versatile for selecting cancer cells that drive metastasis than is the commonly used method of selecting cells from xenografts in nude mice, the latter often being limited by the tissue specificity of cancer cell colonization depending on the site of inoculation.

To determine the potential usefulness of *in vitro-*derived MA cells for testing anticancer agents, we investigated the cells’ molecular and functional characteristics. Our data from gene expression arrays and from comparative genomic hybridization (CGH) arrays revealed several mechanisms in MA cells for generating tumor heterogeneity. These results also revealed that obesity-related molecular pathways, which have evolved to serve a beneficial role in helping organisms survive under harsh metabolic challenges, may be exploited by the rare cancer cells for survival under metabolic challenges. We evaluated the effects of a variety of potential anticancer compounds simultaneously on MA cells and the parental SUM149 cell line. The results validated the MA cells as a good model of panresistance, and that the model can be used for discovering effective anticancer agents.

## Results and Discussion

### Metabolic Adaptability of MA Cells

**Figure 1:**
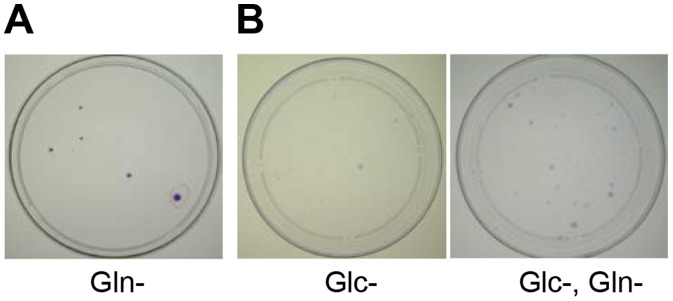
Selection of Rare Cancer Cells That Survive Lack of Glutamine and Glucose. (A) Half a million SUM149-Luc cells were plated in a 10-cm dish. The next day, the medium was changed to a medium containing dialyzed FBS and lacking glutamine. After growth for 34 days in the Gln-deficient medium, we stained the colonies with crystal violet. (B) Half a million SUM149-FP76 cells were plated in a 10-cm dish. The next day, the medium was changed to a medium containing dialyzed FBS lacking glucose (left dish) or lacking both glucose and glutamine (right dish). After 15 days, the media were replaced with complete medium. We stained the colonies with crystal violet after 18 days in complete medium.

The SUM149 cell line is highly dependent on Gln for cell survival and growth. As reported previously, more than 99.99% of SUM149 cells die upon Gln withdrawal [20]. We obtained only 10–20 colonies after plating half a million cells into a 10-cm culture dish (see Fig. 1A for a representative result). In view of the tremendous cellular heterogeneity, it is likely that the 10–20 cells that survived a severe metabolic challenge (prolonged lack of Gln) and yielded colonies are not genomically and epigenomically identical. Significantly, once selected in this manner, MA cells could grow in a Gln-free medium indefinitely. Furthermore, even though initially selected for their ability to survive a lack of Gln, MA cells are also capable of surviving a prolonged lack of glucose [20]. To determine by an alternative approach whether the parental cell line contains rare cells that can survive a lack of both Gln and glucose, we incubated SUM149-FP76 cells (initially cultured from a fat pad xenograft in nude mouse) in Gln- and glucose-deficient medium for 15 days before switching them back to complete medium (all cells eventually die upon glucose withdrawal). We stained the resulting colonies after 18 days, and the staining showed that the number of colonies was not lower than the number of colonies obtained after withdrawal of only glucose (compare the 2 plates in Fig. 1B).

Although some SUM149-Luc cancer cells could survive without glucose or even without glucose and glutamine for approximately 21 days, the experiments involving a lack of glucose are technically more complicated than those involving a lack of glutamine. Since cells can only survive but not grow into colonies without glucose, we must provide glucose to determine the number of surviving cells that form colonies. The number (and presumably the adaptability properties) of surviving cells varies depending upon the number of days glucose was missing (data not shown). In contrast, selection of rare cells surviving the lack of glutamine is easy and reproducible in our system since the surviving cells form colonies within 4–5 weeks. Furthermore, the cells selected in this manner can indefinitely grow in a medium lacking glutamine [20]. Therefore, we chose to focus on SUM149-Luc cells selected in the absence of glutamine for this study.

### Gene Expression and Chromosomal Analysis of MA Cells

**Figure 2:**
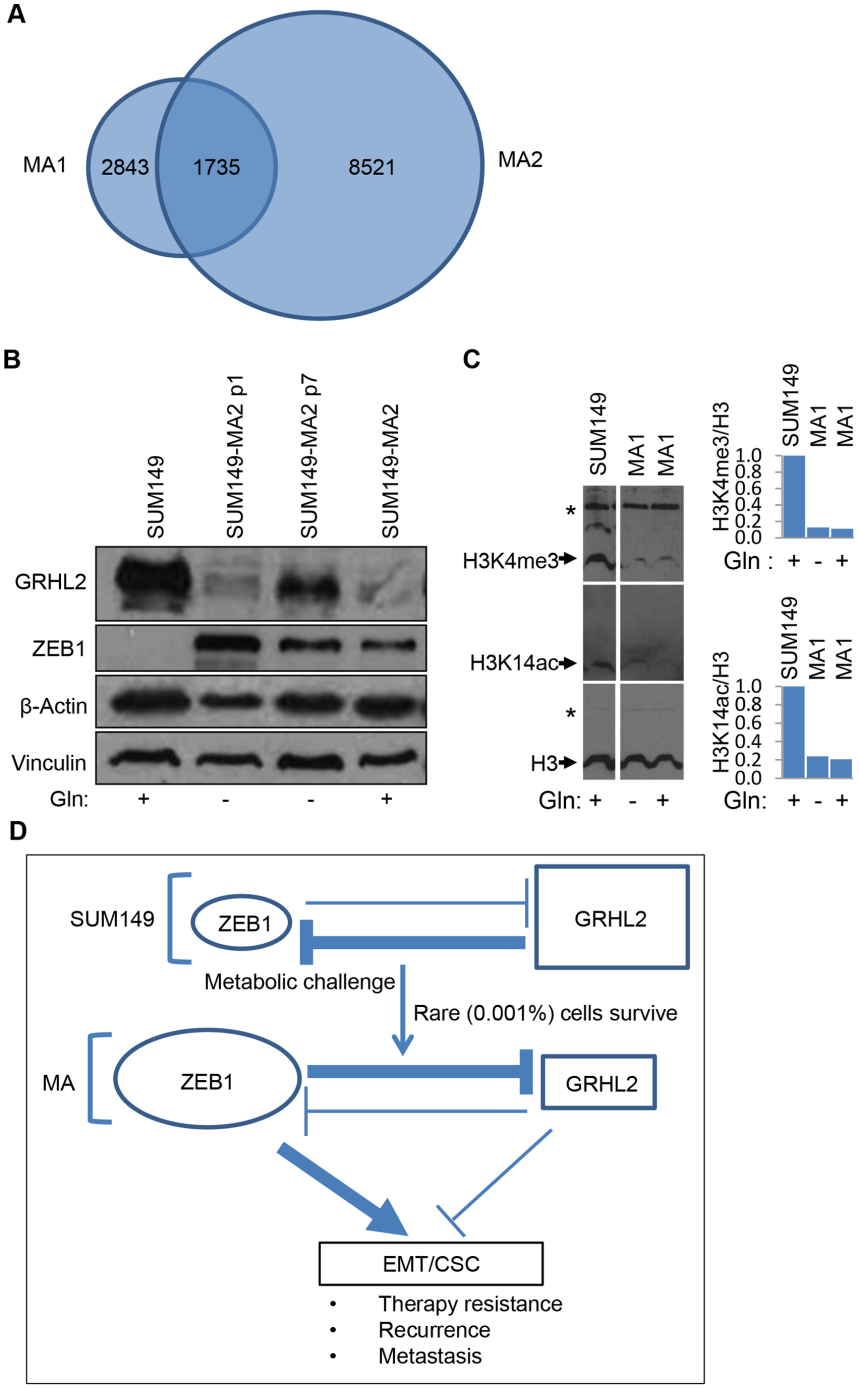
Evidence of a High EMT and a Drug-Tolerant State in MA Cells. (A) Overlap in gene expression changes between MA1 and MA2 variants. The Venn diagram depicts the number of gene probes that detected significantly higher or lower RNA levels in MA1 and MA2 cells than in the parental cell line. The common 1735 gene probes that detected the shared alterations between MA1 and MA2 cells were identified in Microsoft Excel by combining the spreadsheets (Tables S1 and S2) and then searching for duplicate primary sequence names. (B) We analyzed by Western blotting ZEB1 and GRHL2 proteins in MA2 variants and the parental cell line SUM149-Luc (labeled SUM149). After selection in Gln-free medium, MA2 variants were cultured in a medium without or with Gln as indicated at the bottom. p1 and p7 represent passages in Gln-free medium. The parental cell line was cultured in a medium with dialyzed FBS for six passages prior to preparation of lysates for Western blots to match the MA cells. For the rightmost lane, MA2 cells were cultured in Gln-containing medium for four passages prior to preparation of the lysate. We blotted β-actin and vinculin as controls; β-actin is reduced in MA cells grown without Gln, as reported previously [20]. (C) We analyzed by western blotting trimethylation at lysine 4 of histone H3 and acetylation at lysine 14 of histone H3 in MA1 cells and the parental cell line SUM149-Luc (labeled SUM149). We blotted total histone H3 as a control. The star marks a background band that serves as an additional gel loading control in western blots. We cultured MA cells continuously in Gln- medium for 11 passages after selection (Gln-, middle lane), or we cultured MA cells in Gln- medium for 9 passages and then switched them back to Gln+ medium for 4 passages (Gln+, right lane). We measured the relative intensities of histone H3 bands detected as K4me3 and K14ac modified forms and normalized them by dividing with the intensity of total H3 band from the corresponding samples. The normalized data are shown in a graphical form on the right. (D) A model of therapy resistance in MA cells. According to our data, the balance of epithelial versus mesenchymal phenotypes, which is determined by the GRHL2/ZEB1 ratio, is shifted toward mesenchymal phenotype in MA variants. The sizes of ovals and rectangles and the widths of arrows indicate relative levels/strengths. See also Tables S1 and S2.

To gain insight into the characteristic of MA cells that enables them to survive severe metabolic challenge, i.e., prolonged lack of glutamine and other challenges [20], we used gene expression microarrays and CGH arrays to compare these cells with the parental SUM149-Luc (luciferase-transfected) cells. We analyzed two independently selected cell populations, one from 0.5 million parental cells (designated MA1) and one from 1 million parental cells (designated MA2). We generated lists of 2843 gene probes for MA1 cells and 8521 gene probes for MA2 cells that detected significantly altered RNA expression (decreased or increased) relative to RNA expression in the parental cell line (some RNAs were detected with more than one probe) (Tables S1 and S2). Of the significant gene expression changes in MA1 cells, 61% were shared with MA2 cells (Fig. 2A). To gain insight into the significance of the gene expression changes, we performed an Ingenuity Systems pathway analysis. We chose to analyze data from MA2 cells because they had many more changes in gene expression than MA1 cells did. The gene expression profile of significantly altered genes in MA2 cells revealed several associated network functions and molecular and cellular functions that could potentially be important in TNBC (Table S3). Strikingly, network functions with the highest scores included embryonic development, indicating a linkage between metabolic adaptability and overall (embryo-like) adaptability in MA cells. The core analysis of gene expression in MA1 cells also yielded embryonic development as one of the top network functions (not shown). Based on this analysis, the other functions that are likely altered in MA2 cells include lipid metabolism, carbohydrate metabolism, molecular transport, cellular movement, organ development, etc. (Table S3).

We had previously observed that MA cells possess mesenchymal features, including the expression of cadherin 11 and vimentin [20]. On the basis of that finding, we searched for gene expression information for regulators of epithelial-to-mesenchymal transition (EMT) and found that MA cells significantly overexpress zinc finger E-box binding homeobox 1 (ZEB1), a key transcriptional regulator of the mesenchymal phenotype [21]. Furthermore, expression of grainy head-like 2 (GRHL2) RNA, which encodes a transcriptional regulator of epithelial phenotype [21], in MA cells is significantly lower than in the parental cell line (Table S2). We determined by Western blotting that the protein levels of ZEB1 and GRHL2 are consistent with the gene expression data, i.e., ZEB1 protein is up-regulated and GRHL2 protein is down-regulated in MA cells (Fig. 2B). ZEB1 and GRHL2 repress each other, and final phenotype- epithelial or mesenchymal- is dependent on multiple inputs. These results suggest that MA cells possess characteristics of EMT. Cancer cells that have undergone EMT are enriched in cancer stem cell properties and they possess flexible epigenetic state [22], [23]. A high degree of EMT would enable MA cells to generate embryo-like tumor heterogeneity, a prediction supported by the results of gene expression analysis (Table S3).

Our results support a model in which different subpopulations of cancer cells have different levels of metabolic adaptability- minor subpopulations of stem-like non-proliferating cells that can survive severe metabolic challenges and major subpopulations of proliferative, less adaptable non-stem-like cells (Fig. 2D). A severe metabolic challenge, such as the one used for selecting MA cells, which eliminated 99.99% of the cells, selects for highly adaptable, stem-like cancer cells (Fig. 2D). The severity of the metabolic challenge would increase the level of adaptability properties of the cancer cells.

We also examined whether MA cells have a drug-tolerant epigenetic state by measuring methylation and acetylation of histone H3. Significant reductions in both trimethylation at Lys-4 and acetylation at Lys-14 in histone H3 are associated with a drug-tolerant state in cancer [24]. We analyzed the protein modifications by western blotting and quantitated them by measuring band intensities and normalizing them based on equal histone H3 protein in samples. In this manner, we observed that both of these protein modifications were less common in MA cells growing without or with Gln than in the parental cell line (Fig. 2C). These results indicate that MA cells selected in the absence of Gln possess a drug tolerant epigenetic state. Furthermore, the drug tolerant epigenetic state is maintained after the MA cells are switched back to Gln-containing medium (Fig. 2C). These results further support our hypothesis that the adaptability of the rare MA cells may extend beyond metabolism, with drug tolerance being an integral component of this broad adaptability.

**Figure 3:**
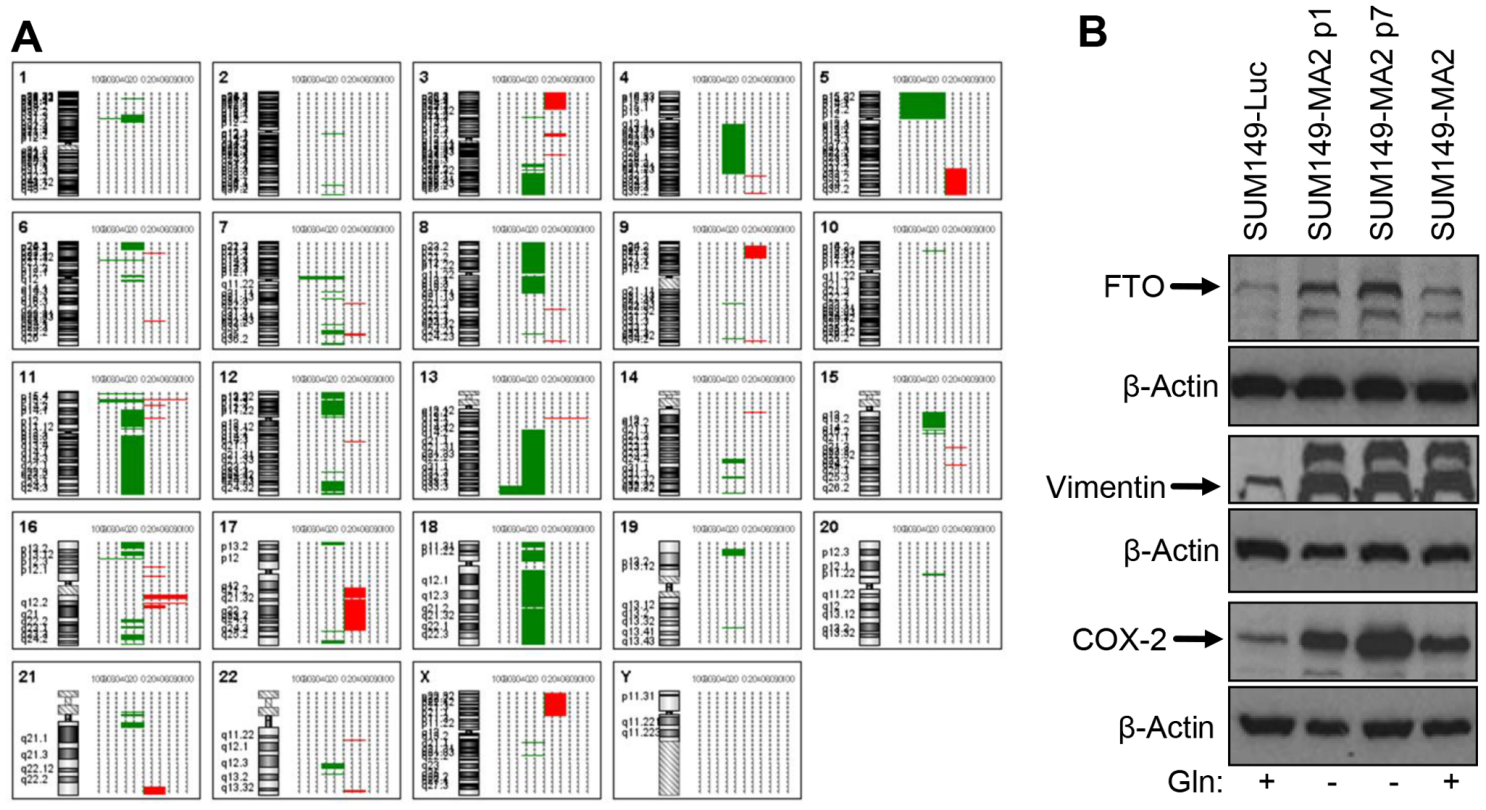
Amplification of the *FTO* Gene in MA Cells. (A) CGH array analysis was performed with MA cells compared to the SUM149-Luc cell line. Chromosomal gains (indicated in red) and losses (indicated in green) in MA1 and MA2 variants are presented as a composite graphic penetrance summary after removal of pseudoautosomal regions from the X and Y chromosomes. Chromosome numbers and the specific loci involved are indicated. Further details are provided in Tables S3 and S4. (B) FTO protein overexpression in MA cells. The cell lysates used in the analysis shown in Fig. 2 were used for these Western blots. The lysates were from the parental cell line, MA2 variants in Gln-free medium at passages 1 and 7, and MA2 variants after 4 passages in Gln-containing medium. We blotted vimentin and COX-2 as controls; we had previously detected their overexpression in MA1/Gln-independent variants [20]. See also Tables S4–S6.

We analyzed chromosomal gains and losses in MA cells as compared to the parental SUM149-Luc cell line by CGH arrays. The CGH array analysis revealed a large number of deletions in MA cells, affecting all chromosomes, some more than others (see Fig. 3A for a graphic summary of chromosomal gains and losses in MA1 and MA2 variants). There were multiple amplifications as well, but amplifications were less frequent than deletions. We also observed that MA1 cells had significantly fewer chromosomal losses and gains than the MA2 cells did (compare Tables S4 and S5). Although some gains and losses were common to MA1 and MA2 cells, the majority of chromosomal changes were not shared by the two cell types (Tables S4–S6). It is not clear at this time whether this difference in MA1 and MA2 cells is due to the difference in input cells, e.g., 0.5 million versus 1 million cells, respectively, for selecting them. Alternatively, some differences could be due to passage number differences between MA1 and MA2 cultures. These results indicate a high degree of chromosomal changes associated with the selection of MA cells. They further suggest that the rare cells selected under a metabolic challenge are likely to be genetically heterogeneous.

Focusing on the chromosomal changes common to MA1 and MA2 cells, we found that the amplified regions involve only three chromosomes: chromosomes 11, 13 and 16 (Table S6). The amplified regions harbor a total of 14 genes (*SBF2, MTUS2, ANKRD26P1, SHCBP1, CBLN1, C16orf78, ZNF423, TMEM188, HEATR3, PAPD5, RBL2, AKTIP, RPGRIP1L,* and *FTO*; see Tables S3–S5 for a list of all genes deleted or amplified in MA1 and MA2 cells and the gene alterations common to MA1 and MA2 cells). The gene that caught our attention in this analysis is *FTO*, one of the four genes (with *RBL2, AKTIP,* and *RPGRIP1L*) amplified on chromosome 16q12.2, for reasons described below.

### *FTO* Gene Amplification in MA Cells

FTO stands for fat mass and obesity-associated protein. It is an alpha-ketoglutarate-dependent dioxygenase belonging to the AlkB family. RNA demethylase activity of FTO is likely to regulate the stability and function of mRNAs [25]. FTO plays an important role in nutrient sensing [26]. It coordinates food intake and consumption in the body. Lack of nutrients, as during starvation, leads to induction of FTO protein in multiple tissues [27]. Certain variants of the *FTO* gene correlate with obesity in humans. These observations suggest that FTO protein plays a key role in organismal survival in nutritionally harsh environments, and inappropriate activation of FTO-mediated protein networks in the absence of metabolic challenges contributes to obesity. Obesity is a commonly observed feature in breast cancer, especially in TNBC and TN-IBC, and may contribute to cancer progression and metastasis [28]. Importantly, genome wide single-nucleotide polymorphism studies strongly suggest that the *FTO* locus is associated with estrogen receptor-negative breast cancer including TNBC but not estrogen receptor-positive breast cancer [29].

Our gene expression microarray data showed that an increased copy number of the *FTO* gene correlated with significantly increased FTO mRNA in MA2 cells (Table S2); this correlation indicates a functional relevance of FTO to the survival of rare (0.01%) SUM149 cells under a severe metabolic challenge. To determine whether the increase in FTO mRNA observed in microarrays led to an increase in the FTO protein level, we analyzed the level of FTO protein by Western blotting. We found that MA cells produced a significantly higher amount of FTO protein than did the parental cell line (Fig. 3B). Interestingly, the level of FTO protein was higher in MA cells growing in a medium without glutamine than in MA cells growing in complete medium with Gln (compare lanes in Fig. 3B). These results are consistent with the results of starvation studies in animals showing that FTO protein is induced upon metabolic challenge. Although MA cells can grow without Gln (albeit slower than they grow in complete medium), we suspect that the cells sense Gln-deficient medium as a nutritionally challenging environment. Our results revealed how obesity and TNBC could be connected at the root level: inappropriate activation or overexpression of FTO could contribute to both obesity and cancer progression, with the latter being orchestrated through the enabling of small subpopulations of cancer cells to survive metabolic challenges in the body. We consider these results highly significant from the perspective of modeling the roots of therapy-resistance in TNBC since MA cells feature genomic abnormalities that are likely relevant in cancer evolution in patients.

### Evidence of Panresistance in MA Cells

**Figure 4:**
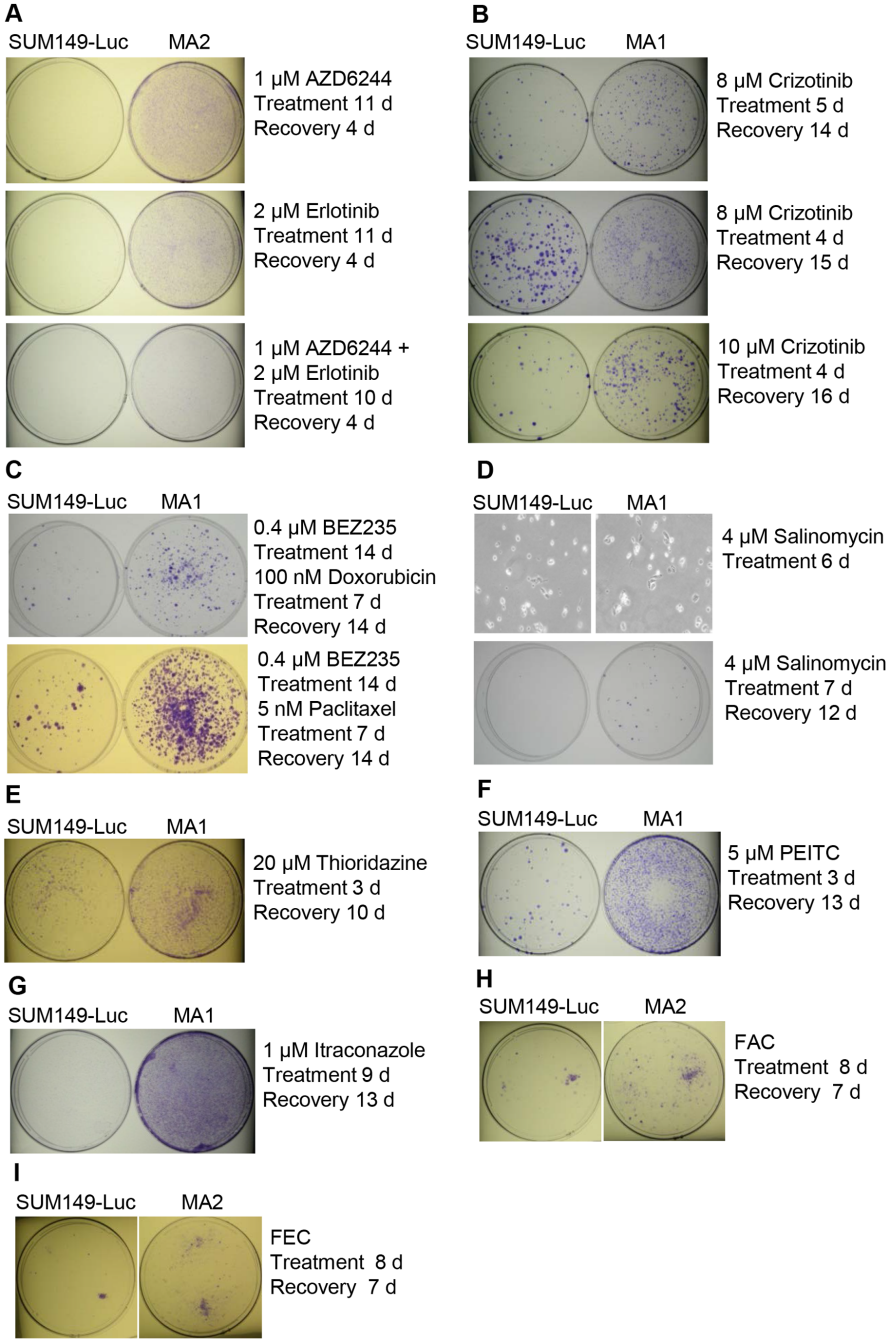
Evidence of an Elevated Panresistance in MA Cells. MA cells and the SUM149-Luc parental cell line were simultaneously treated with various anticancer agents as indicated to determine their relative level of resistance under the conditions that would inhibit most of the proliferating cells. (A) Increased resistance to EGFR and MEK inhibition in MA cells. The cells were treated with erlotinib or AZD6244 for 11 days (d) as indicated (each treatment killed most of the cells) and were allowed to recover in a drug-free medium before the colonies were stained. Combination treatment with the two drugs lasted 10 days, since it killed cells sooner than did the single-drug treatments; then, the cells were allowed to recover for 4 days. (B) Resistance to crizotinib in MA cells. (C) Resistance to PI3K/mTOR inhibition in MA cells. The cells were treated with BEZ235 for 14 days (d), passaged, treated with doxorubicin for 7 days (the treatment killed most of the cells), and allowed to recover and form colonies in a drug-free medium for before the colonies were stained (top). The bottom panel shows a similar experiment in which cells were treated with paclitaxel instead of doxorubicin. (D) Resistance to salinomycin in MA cells. The top panel shows bright-field photographs of representative cells (10X magnification) after 6 days of treatment with salinomycin. The cells were treated with salinomycin for 7 days (which killed most of the cells) and were allowed to recover and form colonies in a drug-free medium before the colonies were stained (bottom panel). (E) Resistance to thioridazine in MA cells. (F) Resistance to PEITC in MA cells. (G) Resistance to itraconazole in MA cells. The parental SUM149-Luc cell line and MA1 cells were treated in parallel with 1 µM itraconazole for 9 days (which killed most of the cells in the parental cell line) and were allowed to recover and grow in a drug-free medium for 5 days before being stained. Since itraconazole was ineffective in killing MA1 cells, the cells grew into a continuous monolayer rather than colonies. (H) Resistance to FAC combination chemotherapy agents in MA cells. (I) Resistance to FEC combination chemotherapy agents in MA cells.

To determine whether MA cells are panresistant, we tested the cells’ responses to a variety of therapeutic agents affecting many cellular processes. We did not solely base our choice of therapeutic agents on the levels of expression of specific targets in MA cells since such information may or may not apply to the roots of resistance (rare, highly adaptable cancer cells). In addition, to focus on the roots of resistance, we chose not to rely on cell proliferation assays. We tested the EGFR inhibitor erlotinib and the MEK inhibitor AZD6244; both targets, EGFR and MEK, are commonly active in TNBC and particularly in the SUM149 cell line [30]. We found that MA cells were more resistant to both of these inhibitors than the parental cell line was. Figure 4A shows representative data obtained with different doses and treatment times. Eleven-day treatment with 2 µM erlotinib or 1 µM AZD6244 was sufficient to eradicate most (>99%) cancer cells in the parental SUM149 cell line. In contrast, a significantly large number of MA cells survived these treatments, as was evident after the treated cells were allowed to recover in drug-free medium for 4 days (compare the stain in left and right panels in Fig. 4A). We also evaluated the effect of combining the two compounds. The combination inhibited MA cells significantly more than did either single agent; however, MA cells remained more resistant than the parental cell line (Fig. 4A, bottom panel). Although the difference in staining is not easily visible in Fig. 4A bottom panel, the staining of MA cells was significantly stronger than was the staining of the parental cell line. Additional experiments using longer recovery time clearly showed that MA cells are more resistant to combination therapy than is the parental cell line (data not shown). Regarding the choice of MA1 versus MA2 cell line for testing therapeutic agents, we have gradually shifted towards using MA2 since it represents a broader heterogeneity (Fig. 2). However, we have tested many therapies, including chemotherapeutic drugs doxorubicin and paclitaxel, on both cell lines and obtained similar results (not shown).

Crizotinib, which is used to treat EML4-ALK-positive non-small cell lung carcinoma, inhibits ALK, MET, and ROS1 tyrosine kinases. These kinases may play a role in breast cancer as well (e.g., see reference [31]). We treated cells with crizotinib for 4–5 days (the treatment killed 99% of the cells) and then allowed the surviving cells to recover and form colonies in drug-free medium for 14–16 days. We observed significantly more colonies in MA cells than in the parental cell line, indicating that MA cells are more resistant to crizotinib than are the parental cells (Fig. 4B).

In view of the fact that elevated PI3K and mTOR activities are common to many cancers including TNBC, BEZ235, a dual PI3K/mTOR inhibitor, is a promising therapeutic agent. A 0.4 µM dose of BEZ235 was ineffective against the very aggressive SUM149 cell line. Therefore, after first treating cells with BEZ235 for 14 days, we treated the cells with doxorubicin for 7 days thus killing >99% of the cells. We allowed the surviving cells to grow into colonies in a drug-free medium for 14 days, after which the cells were visualized by staining (Fig. 4C). MA cells were more resistant to BEZ235/doxorubicin treatment than was the parental cell line. The results indicated that BEZ235 treatment failed to sensitize MA cells to doxorubicin. Similarly, BEZ235 treatment also failed to sensitize MA cells to paclitaxel (Fig. 4C, bottom panel).

Some potentially therapeutic drugs have been identified that would be selective towards cancer stem cells. We reasoned that if there is a significant overlap between the cells selected by our approach and those selected by cancer stem cell approaches, the compounds that are effective against cancer stem cells may be effective against MA cells as well. Consequently, first we evaluated the potential cancer stem cell inhibitor salinomycin [32]. The top panels of Figure 4D show photographs of cells after 6 days of treatment with 4 µM salinomycin (this is how cells typically appear in our experiments after treatment with most test drugs). We found that treatment with 4 µM salinomycin for 7 days eradicated almost all cells in the parental cell line (with the exception of one cell that produced a small colony in drug-free medium after 12 days) , while approximately 40 colonies were observed for MA cells after the cells recovered in a drug-free medium for 12 days (bottom panel in Fig. 4D). This result suggests that the MA cells selected by our approach are resistant to salinomycin.

Thioridazine, a dopamine receptor antagonist that has been identified as an anti-cancer stem cell compound [33], was not very effective in killing SUM149 cells. MA cells were more resistant to 3-day treatment with 20 µM thioridazine than was the parental cell line, as observed after the cells were allowed to recover in a drug-free medium for 10 days (compare the left and right dishes in Fig. 4E). These results suggest that our stringent selection for metabolic adaptability (and co-selection of the embryo-like EMT phenotype) differs from selections that are used to identify cancer stem cells based on surface markers. Nonetheless, the results obtained with the cancer stem cell inhibitors- salinomycin and thioridazine- provide additional evidence of the panresistant nature of MA cells.

One strategy for targeting metastatic cancer cells relies on the observation that these cells suffer from high oxidative stress. It has been proposed that increasing oxidative stress further with therapeutic agents such as PEITC would kill cancer cells without damaging normal cells [34]. Since we had previously observed high oxidative stress in MA cells [20], we tested the efficacy of PEITC against MA cells. We treated cells with 5 µM PEITC for 3 days and allowed the surviving cells to recover and form colonies for 13 days in drug-free medium. We found that PEITC was not very effective against the SUM149 cell line and that it was significantly less effective against MA cells than against the parental cell line. MA cells yielded 10- to 20-fold more colonies than did parental cells (compare the left and right dishes in Fig. 4F), suggesting that the highly adaptable epigenetic state of MA cells enables them to survive various challenges, including high oxidative stress.

Itraconazole is a triazole antifungal drug that is being explored as an anticancer drug for several cancers, e.g., basal cell carcinoma, non-small cell lung cancer, and prostate cancer (e.g., see reference [35]). It inhibits metastasis by antagonizing Smoothened (SMO), thus blocking Hedgehog signaling [36]. Our gene expression microarray analysis indicated SMO overexpression in MA cells. We found that itraconazole was not very effective in eradicating SUM149 cells (Fig. 4G). Furthermore, treatment with 1 µM itraconazole for 9 days followed by 5-day recovery in drug-free medium resulted in significantly more surviving MA cells than surviving parental cells (compare stain intensities, which are proportional to cell masses). The data in Fig. 4G represents a situation wherein treatment with a reasonable dose of a the drug kills most parental cells, but it does not kill MA cells sufficiently to bring down their number in a low range that would yield nicely separated colonies (instead we obtain a lot of cells growing to confluency). This result provides further evidence of the therapy-resistant nature of MA cells.

Finally, we tested combinations of chemotherapeutic agents, formulated as FAC and FEC, which are commonly prescribed for TNBC, particularly for IBC patients. We found that MA cells are more resistant to treatment with FAC (5-fluorouracil, adriamycin, and cyclophosphamide) or FEC (5-fluorouracil, epirubicin, and cyclophosphamide) than the parental cell line (Figs. 4H and 4I), further supporting the utility of MA cells as a cell-based model of therapy-resistant TNBC.

### Strategies for Overcoming Panresistance

Although the question of what would eradicate the roots of the disease, exemplified by the rare MA cells, is difficult to answer, some general guidelines may help in addressing this core problem. Low-dose therapies that are well tolerated (and therefore can be taken for a long period) and that have a low risk of developing resistance to therapy would be preferable to high-dose therapies that are effective against the bulk of the disease (but that do not affect the roots or that may even enrich the roots and thereby lead to therapy resistance). The rationale is that dealing with the roots requires time. One promising approach for eradicating the roots may be the modification of chromatin with the use of agents that alter posttranslational modifications of histones and methylation of DNA. A tremendous amount of new knowledge is emerging regarding regulation of chromatin, leading to discovery of therapeutic agents that alter chromatin. The complexities of chromatin regulation and tumor heterogeneity make it difficult to predict which specific therapy would be optimal for eradicating the roots of TNBC, exemplified by the panresistant MA variants. In a proof-of-principle approach, we began with agents that have been extensively investigated as chromatin modifiers.

**Figure 5:**
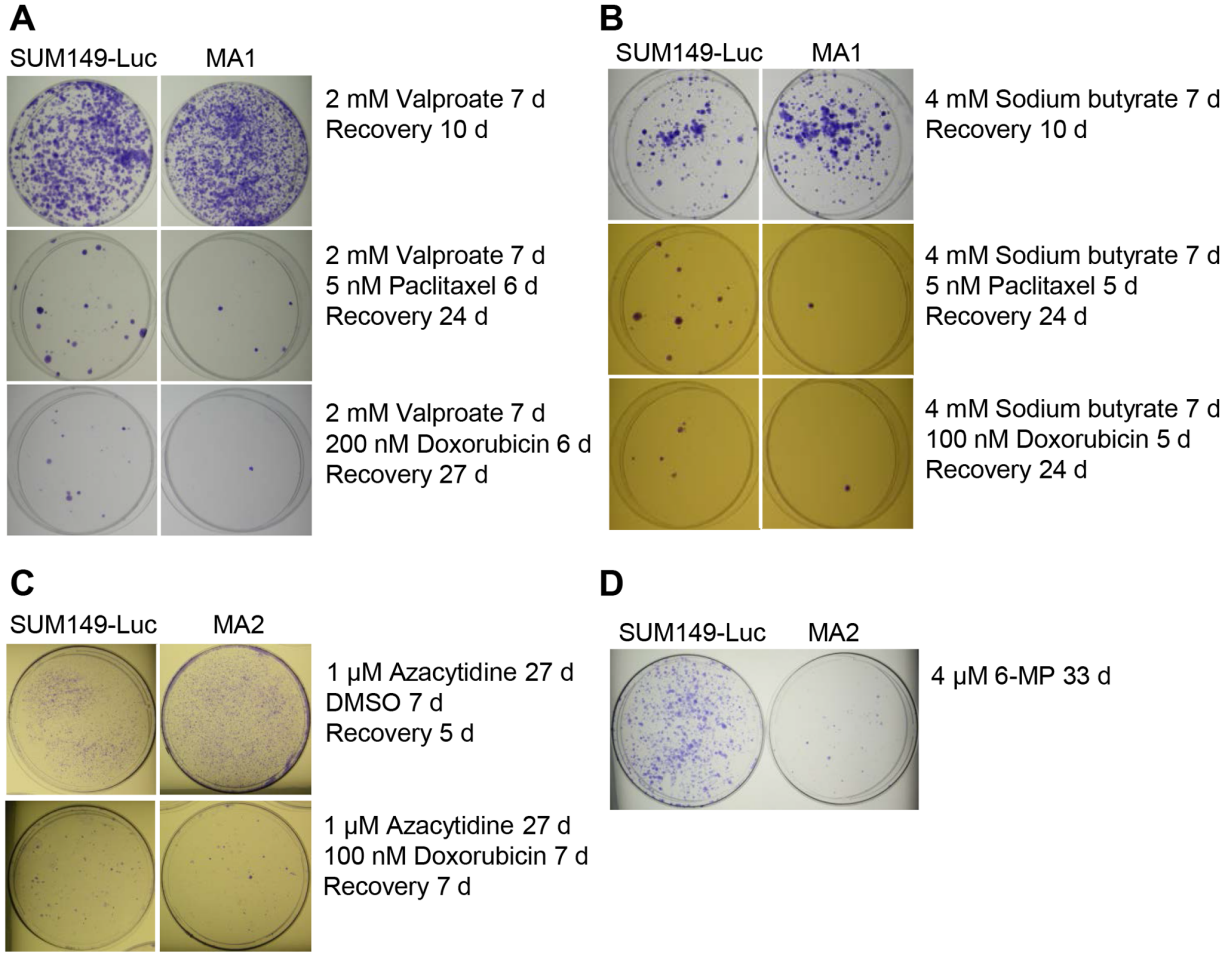
Testing Anticancer Agents for Their Ability to Overcome Panresistance in MA Cells. (A) Sensitization of MA cells to chemotherapeutic drugs by prior treatment with sodium valproate. The parental SUM149-Luc cell line and MA1 cells were treated in parallel with sodium valproate for 7 days (d), passaged, treated with paclitaxel or doxorubicin for 6 days (which killed most of the cells), and allowed to recover and form colonies in a drug-free medium before being stained. (B) Sensitization to chemotherapeutic drugs by prior treatment of MA cells with sodium butyrate. (C) Sensitization to doxorubicin by prior 27-day treatment of MA cells with azacytidine. The cells were treated with azacytidine for 27 days (d) (three passages in culture), passaged, and treated with doxorubicin along with for 7 days (which killed most of the cells), and allowed to recover and form colonies in a drug-free medium for 12 days before being stained (top). Also shown are cells that were treated with the DMSO solvent for 7 days as a control for doxorubicin treatment and that were allowed to recover in a drug-free medium for 5 days prior to being stained (bottom). (D) Eradication of MA cells by 33-day treatment with 6-mercaptopurine. The cells were treated with 6-mercaptopurine for 33 days (three passages in culture) and then stained.

The goal was to determine whether long-term treatment with histone deacetylase (HDAC) inhibitors would sensitize MA variants to chemotherapeutic drugs. HDAC inhibitors are being evaluated against a variety of cancers. Since we observed decreased acetylation at histone H3 lysine 14, which may be part of a drug-tolerant state [24], we evaluated sodium valproate and sodium butyrate, inhibitors of class I and class IIa HDACs [37]. We evaluated them in a low concentration range to minimize cytotoxic effects. We treated cells with various concentrations of the drugs for different periods in duplicate plates and then passaged the cells from subconfluent cultures by trypsinization. The results for cells treated with HDAC inhibitors for 7 days are presented in Fig. 5. After recovery for 1 day, we treated the cells with the chemotherapeutic drug doxorubicin or paclitaxel. When approximately 99% of the cells were killed (after 5–6 days) , we removed the drugs and allowed the cells to recover and form colonies. Representative data from these experiments show that MA cells yielded fewer colonies than did the parental cell line after either valproate-paclitaxel or valproate-doxorubicin treatment (compare plates in Fig. 5A; we obtained a similar results with butyrate-paclitaxel or butyrate-doxorubicin treatment (Fig. 5B). Given the therapy-resistant nature of MA cells, even equal numbers of colonies in MA cells and the parental cell line would indicate sensitization of MA cells to chemotherapy. Thus, our results indicate that both valproate and butyrate effectively sensitize MA cells to chemotherapeutic drugs.

A low-dose 5-azacytidine treatment leads to reduced DNA methylation. Azacytidine and its deoxy derivative are used for treating Myelodysplastic Syndromes, and they are being evaluated in clinical trials in various combination therapies for several additional cancers. Here, we investigated whether 5-azacytidine treatment can sensitize MA cells to chemotherapeutic drugs. Since reversal of epigenetic modifications may require multiple cell divisions, we treated cells with azacytidine for different periods ranging from 7 days (one passage) to 27 days (three passages). Although initially 1 µM azacytidine was not cytotoxic as evidenced by a lack of significant growth-inhibition, we noticed that after 27 days of treatment the parental cell line had approximately 50% fewer cells than did the MA2 cell line. At that time, we passaged the cells while continuing to treat them with azacytidine in combination with doxorubicin for 7 days; the combination treatment killed >99% of the cells. After allowing the surviving cells to recover and form colonies in a drug-free medium for 12 days, we stained the colonies (Fig. 5C). We found that azacytidine treatment resulted in significant sensitization of MA cells to doxorubicin: the number of doxorubicin-resistant colonies among MA cells was approximately the same as or lower than the number of doxorubicin-resistant colonies formed by the parental cell line (Fig. 5C). Given that without prior treatment, doxorubicin-treated MA cells yield significantly (5–10-fold) more colonies than the parental cell line does [20], our results indicate that azacytidine may help overcome resistance to chemotherapeutic drugs in MA cells.

We hypothesized that 6-mercaptopurine, which is used to treat leukemia and several other diseases, including inflammatory bowel disease, may help overcome resistance of MA cells to anticancer agents. What makes 6-mercaptopurine attractive for our purpose is the safety and efficacy of low-dose 6-MP regimens administered over many years. We treated MA cells and the parental cell line in parallel with 4 µM 6-mercaptopurine for 33 days continuously, involving three passages in cell culture. 6-Mercaptopurine did not cause any appreciable cytotoxicity in either cell line during the first two passages. However, it had a dramatically greater effect on MA cells than on the parental cell line during the third passage. Colonies stained after 17 days of growth in passage 3 in the presence of 6-mercaptopurine are shown in Fig. 5D. A significantly lower number of colonies from MA cells than the parental cell line indicates that long-term treatment with 6-mercaptopurine may have exhausted MA cells.

In view of the evolution-like nature of cancer, it is critical that we get better at predicting evolution of cancer as therapies are implemented. The therapies would influence cancer evolution and often rare clones that are relatively indolent at the time of diagnosis would assume an active role after therapeutic interventions [1], [38], [39]. Although it is not feasible to model the cancer evolution that occurs in individual patients, the strategy presented here can assist in modeling the roots of cancer that are likely to be relevant in continuously evolving cancer. An important lesson from our study is that to get most out of a cell culture model we must constantly ask what would be the likely fate of the specific subpopulations of cancer cells in cell culture when such cells are part of a heterogeneous tumor evolving in the body, particularly under currently offered therapeutic interventions. Addressing this question would be a basis of selecting adaptable cancer cells and for selecting/designing assays that inform about adaptable cells even though the cell culture conditions do not impose body-like selection pressures.

Based on our results, we believe that *FTO* gene amplification leading to higher FTO mRNA and protein level helped MA cells to survive a severe metabolic challenge in the form of lack of glutamine. Among other important elements in this selection may be the embryo-like nature of rare cells that survived. Based on work with whole animals and with cell lines, FTO is important in starvation response [27], [40]. We found that the level of FTO protein begins to come down when cells are cultured in complete medium (Fig. 3B). We interpret these data to suggest that a higher level of FTO protein may not be required or may not be beneficial for cell growth in complete medium. By modulating the function of many mRNAs and non-coding RNAs through demethylation, FTO is an important part of circuitry that controls metabolism at cellular and organismal levels. Investigating the functional role of FTO in MA cells and how to therapeutically exploit it for cancer treatment may be fruitful. Besides the complexity of obesity networks involving FTO deregulation, the major interconnected problem in disease like TNBC is tumor heterogeneity and tumor adaptability.

There is significant interest in glutamine metabolism in cancer. Glutamine serves as an important carbon and nitrogen source for biosynthetic processes in cancer; it is also important in regulating redox status. As far as studies pertaining to metabolic challenges are concerned, a major effort has been to investigate the consequences of hypoxia in tumors. However, it is apparent that tumors not only lack oxygen in some regions, they also lack other nutrients, e.g., glutamine [41]. Such metabolic challenges are likely to influence tumor evolution. Studies have also been carried out to determine the effect of Gln withdrawal *in vitro*. However, unlike this study that aims to isolate metabolically adaptable rare cancer cells, those studies have mostly investigated the effect of Gln withdrawal on the major cancer cell population (e.g., see reference 41).

**Figure 6:**
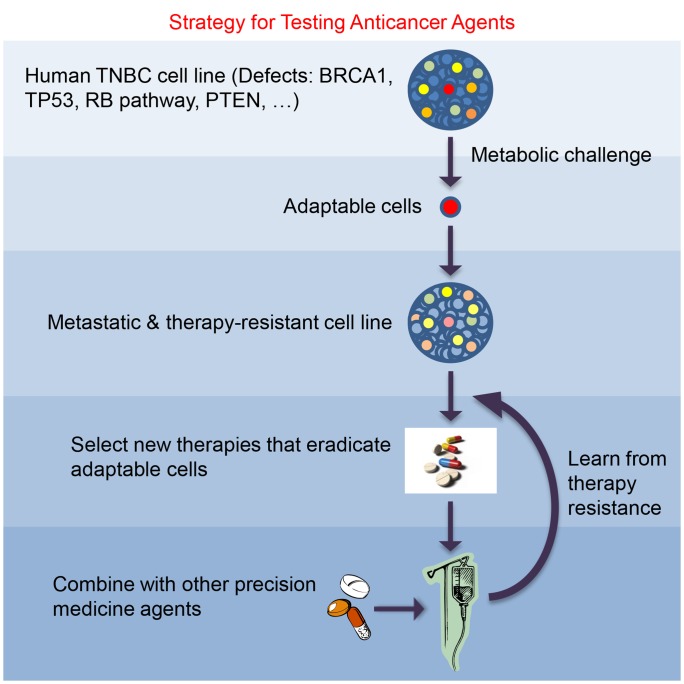
A Strategy for Testing Anticancer Agents. The strategy described in this paper is depicted as a flow diagram. See text for further details.

We have summarized our strategy for modeling the roots of TNBC, and for testing anticancer agents in Figure 6. Although our approach has unique features that make it useful for testing anticancer agents, we also recognize the limitations of the approach. Even though our strategy is good at modeling the roots of panresistance, all *in vitro* approaches including ours have a drawback of lacking suitable microenvironments that cancer cells encounter in the body. Although one can choose from a variety of techniques aimed at modeling a tumor microenvironment in cell culture, such techniques have severe limitations. Broadly speaking, modeling appropriate tumor environment is not easy since tumor cells face a variety of microenvironments in the body. In this study, our main goal was to investigate the most adaptable rare cells for their potential utility in anticancer drug testing. We recognize that all models of cancer have different sets of limitations. Therefore, unique strengths of different models, including that of ours, need to be utilized for developing effective therapies against cancer.

### Conclusions

To summarize, we have a suitable model of hard-to-eradicate roots for testing anticancer therapies. Our approach can be used to test a growing list of potential therapeutic compounds that can target the majority of cancer cells through a variety of mechanisms. The cancer cells most responsible for panresistance are heterogeneous, and the likelihood that a particular test therapy will affect these cells is difficult to predict. Our testing strategy is optimized to investigate the effects of potential therapeutic compounds on such cells. Our *in vitro* approach can complement other approaches, including therapy testing in genetically engineered mouse models and patient-derived cancer xenografts in mice. Since evaluation of combination therapies (drugs, doses, schedules, etc.) is much easier in cell lines than in mice, our *in vitro* strategy can be used to merge realistic function-based selection of cancer cells most responsible for therapy resistance with the common biomarker/driver-based drug discovery approach to develop therapies that can overcome resistance. Our approach can be used to test sensitizing agents (that may alter root cells when administered at a low dose over a long period) and inhibitors of molecular drivers of disease (whose range of target cells may broaden as a result of prior treatment with sensitizing agents) and to identify therapies that are effective in combating disease recurrence and metastasis and that are suitable for clinical trials. If aligned with the clinic, this approach can not only help choose superior therapies for clinical trials, but also suggest strategies for overcoming therapeutic resistance in a timely manner.

## Materials and Methods

### Cell Lines and Culture

The SUM149 IBC cell line, originally obtained from Stephen Ethier (Barbara Ann Karmanos Cancer Institute, Detroit, MI, USA), was grown in Ham’s F-12 medium supplemented with 5% fetal bovine serum (FBS), 5 µg/ml of insulin, 1 µg/ml of hydrocortisone, 100 U/ml of penicillin, and 100 µg/ml of streptomycin in a humidified 5% CO_2_ atmosphere. We previously described SUM149-Luc, a luciferase-transfected cell line [17]. SUM149-FP76 is another cell line recently developed in our laboratory by first culturing cells from a SUM149-Luc xenograft subcutaneously in a female nude mouse and then culturing cells from a fat pad xenograft [20].

### Selection and Culture of MA Variants

We selected MA variants by plating 0.5 million or 1 million SUM149-Luc cells in a glutamine-deficient medium containing dialyzed FBS. We recently described selection of rare variants (13 colonies from 0.5 million cells) and initial characterization of a cell culture established from these colonies [20]. In the current study, we refer to this cell line as MA1, and we refer to another cell line that was similarly established by selecting variants from 1 million cells in a glutamine-deficient medium as MA2. Although MA1 and MA2 cells can be passaged indefinitely in glutamine-deficient medium, we cultured them in glutamine-containing medium for testing therapeutic agents. Both MA variants and the parental SUM149-Luc cell line were cultured identically in regular medium containing dialyzed FBS for this purpose. We performed all experiments with the cell lines before passage 10 in this medium. We also cultured MA cells in a glutamine-deficient medium in some experiments as indicated; the purpose of these experiments was to determine whether the continued absence of glutamine affected the phenotype under investigation.

### Gene Expression Microarray Analysis

For gene expression microarray analysis, we cultured both MA variants and the parental cell line in a medium containing Gln and dialyzed FBS; we used dialyzed FBS since it was used for selecting the MA variants in Gln-free medium. RNA was isolated using standard RNA extraction protocols (NucleoSpin RNA II, Macherey-Nagel, Bethlehem, PA) and quality checked with the 2100 Bioanalyzer (Agilent Technologies). To produce cyanine 3-labeled complementary RNA, the 100-ng RNA samples were amplified by linear T7 polymerase-based amplification and labeled using the Agilent Low Input Quick Amp Labeling Kit according to the manufacturer’s protocol. Hybridization was performed according to the Agilent 60-mer oligo microarray processing protocol using the Agilent Gene Expression Hybridization Kit. Briefly, 600 ng of cyanine 3-labeled fragmented complementary RNA in hybridization buffer was hybridized overnight (17 hours, 65°C) to Agilent Whole Human Genome Oligo 8×60K Microarrays. The fluorescence signals of the hybridized microarrays were detected using the Agilent Microarray Scanner System. Agilent G2567AA Feature Extraction Software version 10.7.3.1 was used to read out and process the microarray image files. The software determines feature intensities (including background subtraction), rejects outliers, and calculates statistical confidences. To determine differential gene expression, Feature Extraction Software-derived output data files were further analyzed using the Rosetta Resolver gene expression data analysis system (Rosetta Biosoftware). Putative candidate genes with fold changes >2 and p-values <0.01 are summarized in the preselected candidate genes lists.

### CGH Array Analysis

For CGH array analysis, cells were cultured in parallel with those used for the gene expression microarray. Genomic DNA was purified using the Gentra Puregene Cell kit (Qiagen). The integrity of the genomic DNA samples was assessed by agarose gel electrophoresis. A 2.5-µg quantity of each DNA sample was digested with the restriction enzymes *Alu*I and *Rsa*I. The digested DNA was used as template for a genomic DNA labeling reaction using the SureTag DNA Labeling Kit (Agilent Technologies) according to the manufacturer’s protocol. The yields of labeled DNA and the dye incorporation rate after Klenow labeling were determined with the ND-1000 Spectrophotometer (NanoDrop Technologies). Hybridization was performed according to the Agilent Oligonucleotide Array-Based CGH for Genomic DNA Analysis protocol version 7.1 using the SureTag DNA Labeling Kit. The corresponding cyanine 3- and cyanine 5-labeled DNAs were combined and hybridized at 65°C for 40 hours to an Agilent Human Genome CGH 1M Microarray. The fluorescence signals of the hybridized Agilent Human Genome CGH microarrays were detected using the Agilent DNA microarray scanner. Agilent Feature Extraction Software was used to read out and process the microarray image files. Further analysis and visualization of the hybridization results were performed with the Agilent Genomic Workbench Lite 6.5. We used the following stringent filter settings for aberrations: (i) minimum number of probes present in an aberrant region = 5, (ii) minimum absolute average log2 ratio for region = 0.4 (corresponds to a fold change of 1.32), and (iii) threshold = 5. The statistical analysis of aberrant regions was based on the aberration detection method ADM-2. The ADM-2 algorithm identifies all aberrant intervals in a genome with consistently high or low log ratios based on the statistical score.

### Western Blotting

We separated proteins by sodium dodecyl sulfate-polyacrylamide gel electrophoresis and detected various proteins by Western blotting, as described previously [42]. The following primary antibodies were used for detection: anti-COX-2 monoclonal antibody (Cayman Chemical, Ann Arbor, MI), anti-GRHL2 (Sigma-Aldrich, St. Louis, MO), anti-FTO (EMD Millipore, Billerica, MA), anti-ZEB1, anti-histone H3, anti-histone H3 tri-methyl Lys-4, anti-histone H3 acetyl Lys-14, and anti-vimentin antibodies (Cell Signaling, Danvers, MA). We used the ECL prime blocking agent (GE Healthcare Life Sciences, Piscataway, NJ) for blocking and Lumigen TMA-6 reagents for detection (Lumigen, Inc., Southfield, MI). The filters were stripped by incubating the membrane in 0.5% Triton X-100 and were re-probed with a monoclonal β-actin antibody (Sigma-Aldrich, St. Louis, MO) or with vinculin antibody (Abcam, Cambridge, MA), which served as gel-loading controls. To detect β-actin or vinculin, we used 2% non-fat dry milk (Bio-Rad, Hercules, CA) for blocking and ECL prime reagent for detection (GE Healthcare Life Sciences, Piscataway, NJ). We performed each western blot at least twice; the representative blots are shown. We quantified the protein bands on x-ray films by using the ImageJ image processing program (National Institutes of Health).

### Metabolic Adaptability Assay

The basis of the metabolic adaptability assay is that prolonged extreme glucose deficiency is likely to select cells with a highly adaptable metabolic state. Cancer cells are highly addicted to glucose, so the majority of them die within a few days in the absence of glucose; in fact, a prolonged lack of glucose eventually kills all cancer cells in culture. To determine the metabolic adaptability of MA cells, we deprived them of glucose for 2 to 4 weeks and assessed whether a higher number of cells survived than the number of parental cells that survived glucose deprivation. We plated a half million cells on a 10-cm dish, switched them to glucose-free medium (custom medium from AthenaES, Baltimore, MD) the next day for 2 to 4 weeks, and then switched them back to complete medium for 2–4 weeks until the surviving cells yielded colonies. We stained the colonies with crystal violet and photographed them. In some experiments, we deprived cells of both glucose and glutamine and compared the surviving cells with those selected by glucose deprivation alone.

### Test Drugs

We purchased the drugs from the commercial sources: paclitaxel, doxorubicin/adriamycin, 5-fluorouracil, epirubicin, cyclophosphamide, crizotinib, salinomycin, thioridazine, phenethyl isothiocyanate (PEITC), sodium valproate, sodium butyrate, 5-azacytidine, and 6-mercaptopurine (Sigma-Aldrich, St. Louis, MO), NVP-BEZ235 (Cayman Chemical, Ann Arbor, MI), and itraconazole (Selleckchem, Houston, TX). Naoto Ueno kindly provided AZD6244 (AstraZeneca, Wilmington, DE) and erlotinib (ChemieTek, Indianapolis, IN). We dissolved BEZ235, PEITC, itraconazole, crizotinib, salinomycin, doxorubicin, paclitaxel, AZD6244, and erlotinib in dimethyl sulfoxide (DMSO), thioridazine, sodium valproate, and sodium butyrate in water, 6-mercaptopurine in 0.1 M NaOH, and 5-azacytidine in dulbecco’s phosphate buffered saline. To prepare FAC (5-fluorouracil, adriamycin, cyclophosphamide) and FEC (5-fluorouracil, epirubicin, cyclophosphamide) chemotherapy combinations, drugs were measured individually and dissolved into DMSO and combined. Final concentrations of drugs were 250 nM 5-fluorouracil, 25 nM adriamycin, and 250 nM cyclophosphamide for FAC and 250 nM 5-fluorouracil, 50 nM epirubicin, and 250 nM cyclophosphamide for FEC. We added equal volume of the solvent in all dishes including the control dishes without drugs. DMSO volume was ≤0.04% of the volume of the culture medium.

### Evaluation of Test Drugs *in vitro*

In a comparative long-term evaluation of therapeutic agents, in which >99% of the cells in the parental cell line were killed, we determined the number of MA cells that survived and yielded colonies. Since it is often not easy to determine that therapeutic agents has killed more than 99% of the cells in a culture dish, we used different drug concentrations and treatment times to improve the likelihood of detecting this result. We typically plated 0.5 million cells per 10-cm dish in duplicate in culture medium with glutamine. After 24 hours, we added a test drug at four different concentrations (1X, 2X, 4X, and 8X) based on literature in a preliminary experiment to determine the drug concentration range that would eradicate >99% cells in about a week. The cells were photographed, and plates were monitored for different periods until most cells (>99%) were killed. To compare the cell survival rates in the MA cells and the SUM149 cell line, drugs were removed by rinsing twice with phosphate-buffered saline, and cells were maintained in a drug-free medium for 3–4 weeks until colonies were visible to the naked eye. Colonies were stained with crystal violet and photographed.

Because some therapeutic agents that are ineffective at killing cancer cells may nevertheless sensitize the cells to other chemotherapeutic drugs, we evaluated combination therapies, wherein two agents were added concurrently or sequentially to MA cells or the parental cell line. In evaluating therapies for their potential to sensitize MA cells to chemotherapeutic drugs, some experiments involved treatment that lasted several weeks, which made it necessary to passage the cell cultures.

### Accession Numbers

The gene expression microarray data and CGH array data discussed in this publication have been deposited in NCBI's Gene Expression Omnibus and are accessible through GEO Series accession number GSE60017 (http://www.ncbi.nlm.nih.gov/geo/query/acc.cgi?acc=GSE60017).

## Supporting Information

Table S1 **Genes Encoding Significantly Altered RNA Expression in MA1 Cells, Related to** **Figure 2** **.** We generated a list of genes with significantly altered RNA levels in MA1 cells relative to the RNA levels in the parental cell line as described in Extended Experimental Procedures. The genes are listed in order from the most reduced RNA to the most increased RNA, with fold changes in RNA level and p-values indicated. The MA1 cells were passaged 11 times in glutamine-free medium followed by 5 times in glutamine-containing medium prior to this analysis.
(XLSX)

Table S2 **Genes Encoding Significantly Altered RNA Expression in MA2 Cells, Related to** **Figure 2** **.** We generated a list of genes with significantly altered RNA levels in MA2 cells relative to the RNA levels in the parental cell line as described in Extended Experimental Procedures. The genes are listed in order from the most reduced RNA to the most increased RNA, with fold changes in RNA level and p-values indicated. The MA2 cells were passaged 10 times in glutamine-free medium followed by 3 times in glutamine-containing medium prior to this analysis.
(XLSX)

Table S3 **Molecular Alterations Affecting Several Networks in MA2 cells, Related to** **Figure 2** **.** The significant alterations in gene expression in MA2 cells, which are listed in Table S2, were subjected to core analysis in the Ingenuity Pathway Analysis software. Significantly up-regulated or down-regulated molecules are grouped according to the diseases and functions they may impact. The analysis is composed of 25 networks.
(PDF)

Table S4 **Chromosomal Gains and Losses in MA1 Cells, Related to** **Figure 3** **.** The 42 aberrations detected under stringent settings are listed. The list includes all the genes located in the affected chromosomal regions.
(XLS)

Table S5 **Chromosomal Gains and Losses in MA2 Cells, Related to** **Figure 3** **.** The 283 aberrations detected under stringent settings are listed. The list includes all the genes located in the affected chromosomal regions.
(XLS)

Table S6 **Chromosomal Gains and Losses Shared by MA1 and MA2 Cells, Related to** **Figure 3** **.** The 13 aberrations (5 amplifications and 8 deletions) detected under stringent settings in both MA1 cells and MA2 cells are listed. The list includes all the genes located in the affected chromosomal regions. We extracted this information manually in Microsoft Excel from Tables S4 and S5.
(DOCX)

## References

[1] Nowell PC (1976) The clonal evolution of tumor cell populations. Science 194: 23–28. 959840 10.1126/science.959840

[2] Fidler IJ, Kripke ML (1977) Metastasis results from preexisting variant cells within a malignant tumor. Science 197: 893–895. 887927 10.1126/science.887927

[3] Talmadge JE, Wolman SR, Fidler IJ (1982) Evidence for the clonal origin of spontaneous metastases. Science 217: 361–363. 6953592 10.1126/science.6953592

[4] Chambers AF, Groom AC, MacDonald IC (2002) Dissemination and growth of cancer cells in metastatic sites. Nat Rev Cancer 2: 563–572. 12154349 10.1038/nrc865

[5] Talmadge JE, Fidler IJ (2010) AACR centennial series: the biology of cancer metastasis: historical perspective. Cancer Res 70: 5649–5669. 20610625 10.1158/0008-5472.CAN-10-1040 PMC4037932

[6] Cifone MA, Fidler IJ (1980) Correlation of patterns of anchorage-independent growth with *in vivo* behavior of cells from a murine fibrosarcoma. Proc Natl Acad Sci U S A 77: 1039–1043. 6928659 10.1073/pnas.77.2.1039 PMC348419

[7] Al-Hajj M, Wicha MS, Benito-Hernandez A, Morrison SJ, Clarke MF (2003) Prospective identification of tumorigenic breast cancer cells. Proc Natl Acad Sci U S A. 100: 3983–3988. 10.1073/pnas.0530291100 PMC153034 12629218

[8] Guo L, Fan D, Zhang F, Price JE, Lee JS, et al (2011) Selection of brain metastasis-initiating breast cancer cells determined by growth on hard agar. Am J Pathol 178: 2357–2366. 21514446 10.1016/j.ajpath.2011.01.047 PMC3081177

[9] McKnight SL (2010) On getting there from here. Science 330: 1338–1339. 21127243 10.1126/science.1199908

[10] Shah SP, Roth A, Goya R, Oloumi A, Ha G, et al (2012) The clonal and mutational evolution spectrum of primary triple-negative breast cancers. Nature 486: 395–399. 22495314 10.1038/nature10933 PMC3863681

[11] The Cancer Genome Atlas Network (2012) Comprehensive molecular portraits of human breast tumours. Nature 490: 61–70. 23000897 10.1038/nature11412 PMC3465532

[12] Zhang Q1, Liu Y, Zhou J, Chen W, Zhang Y, et al (2007) Wild-type p53 reduces radiation hypermutability in p53-mutated human lymphoblast cells. Mutagenesis. 22: 329–334. 10.1093/mutage/gem021 17567629

[13] Bullock AN, Henckel J, Fersht AR (2000) Quantitative analysis of residual folding and DNA binding in mutant p53 core domain: definition of mutant states for rescue in cancer therapy. Oncogene 19: 1245–1256. 10713666 10.1038/sj.onc.1203434

[14] Chao HH, He X, Parker JS, Zhao W, Perou CM (2012) Micro-scale genomic DNA copy number aberrations as another means of mutagenesis in breast cancer. PLoS One. 7: e51719. 10.1371/journal.pone.0051719 PMC3524128 23284754

[15] Barnabas N, Cohen D (2013) Phenotypic and molecular characterization of MCF10DCIS and SUM breast cancer cell lines. Int J Breast Cancer 2013: 872743. 23401782 10.1155/2013/872743 PMC3562669

[16] Pan Q, Bao LW, Merajver SD (2003) Tetrathiomolybdate inhibits angiogenesis and metastasis through suppression of the NFkappaB signaling cascade. Mol Cancer Res 1: 701–706. 12939395

[17] Singh B, Cook KR, Martin C, Huang EH, Mosalpuria K, et al (2010) Evaluation of a CXCR4 antagonist in a xenograft mouse model of inflammatory breast cancer. Clin Exp Metastasis 27: 233–240. 20229045 10.1007/s10585-010-9321-4

[18] Van Laere SJ, Ueno NT, Finetti P, Vermeulen P, Lucci A, et al (2013) Uncovering the molecular secrets of inflammatory breast cancer biology: an integrated analysis of three distinct affymetrix gene expression datasets. Clin Cancer Res. 19: 4685–4696. 10.1158/1078-0432.CCR-12-2549 PMC6156084 23396049

[19] Masuda H, Baggerly KA, Wang Y, Iwamoto T, Brewer T, et al (2013) Comparison of molecular subtype distribution in triple-negative inflammatory and non-inflammatory breast cancers. Breast Cancer Res. 15: R112. 10.1186/bcr3579 PMC3978878 24274653

[20] Singh B, Tai K, Madan S, Raythatha MR, Cady AM, et al (2012) Selection of metastatic breast cancer cells based on adaptability of their metabolic state. PLoS ONE 7: e36510. 22570721 10.1371/journal.pone.0036510 PMC3343010

[21] Cieply B, Farris J, Denvir J, Ford HL, Frisch SM (2013) Epithelial-mesenchymal transition and tumor suppression are controlled by a reciprocal feedback loop between ZEB1 and Grainyhead-like-2. Cancer Res. 73: 6299–6309. 10.1158/0008-5472.CAN-12-4082 PMC3806457 23943797

[22] Mani SA, Guo W, Liao MJ, Eaton EN, Ayyanan A, et al (2008) The epithelial-mesenchymal transition generates cells with properties of stem cells. Cell. 133: 704–715. 10.1016/j.cell.2008.03.027 PMC2728032 18485877

[23] Tam WL1, Weinberg RA (2013) The epigenetics of epithelial-mesenchymal plasticity in cancer. Nat Med. 19: 1438–1449. 10.1038/nm.3336 PMC4190672 24202396

[24] Sharma SV, Lee DY, Li B, Quinlan MP, Takahashi F, et al (2010) A chromatin-mediated reversible drug-tolerant state in cancer cell subpopulations. Cell 141: 69–80. 20371346 10.1016/j.cell.2010.02.027 PMC2851638

[25] Gerken T, Girard CA, Tung YC, Webby CJ, Saudek V, et al (2007) The obesity-associated FTO gene encodes a 2-oxoglutarate-dependent nucleic acid demethylase. Science 318: 1469–1472. 17991826 10.1126/science.1151710 PMC2668859

[26] Gulati P, Cheung MK, Antrobus R, Church CD, Harding HP, et al (2013) Role for the obesity-related FTO gene in the cellular sensing of amino acids. Proc Natl Acad Sci U S A. 110: 2557–2562. 10.1073/pnas.1222796110 PMC3574930 23359686

[27] Fredriksson R, Hägglund M, Olszewski PK, Stephansson O, Jacobsson JA, et al (2008) The obesity gene, FTO, is of ancient origin, up-regulated during food deprivation and expressed in neurons of feeding-related nuclei of the brain. Endocrinology. 149: 2062–2071. 10.1210/en.2007-1457 18218688

[28] Jain R, Strickler HD, Fine E, Sparano JA (2013) Clinical studies examining the impact of obesity on breast cancer risk and prognosis. J. Mammary Gland Biol. Neoplasia 18: 257–266. 10.1007/s10911-013-9307-3 24221746

[29] Garcia-Closas M, Couch FJ, Lindstrom S, Michailidou K, Schmidt MK, et al (2013) Genome-wide association studies identify four ER negative-specific breast cancer risk loci. Nat Genet. 45: 392–398. 10.1038/ng.2561 PMC3771695 23535733

[30] Zhang D, LaFortune TA, Krishnamurthy S, Esteva FJ, Cristofanilli M, et al (2009) Epidermal growth factor receptor tyrosine kinase inhibitor reverses mesenchymal to epithelial phenotype and inhibits metastasis in inflammatory breast cancer. Clin Cancer Res 15: 6639–6648. 19825949 10.1158/1078-0432.CCR-09-0951 PMC2783487

[31] Raghav KP, Wang W, Liu S, Chavez-MacGregor M, Meng X, et al (2012) cMET and phospho-cMET protein levels in breast cancers and survival outcomes. Clin. Cancer Res. 18: 2269–2277. 10.1158/1078-0432.CCR-11-2830 PMC3821167 22374333

[32] Gupta PB, Onder TT, Jiang G, Tao K, Kuperwasser C, et al (2009) Identification of selective inhibitors of cancer stem cells by high-throughput screening. Cell 138: 645–659. 19682730 10.1016/j.cell.2009.06.034 PMC4892125

[33] Sachlos E, Risueño RM, Laronde S, Shapovalova Z, Lee JH, et al (2012) Identification of drugs including a dopamine receptor antagonist that selectively target cancer stem cells. Cell 149: 1284–1297. 22632761 10.1016/j.cell.2012.03.049

[34] Trachootham D, Zhou Y, Zhang H, Demizu Y, Chen Z, et al (2006) Selective killing of oncogenically transformed cells through a ROS-mediated mechanism by beta-phenylethyl isothiocyanate. Cancer Cell 10: 241–252. 16959615 10.1016/j.ccr.2006.08.009

[35] Rudin CM, Brahmer JR, Juergens RA, Hann CL, Ettinger DS, et al (2013) Phase 2 study of pemetrexed and itraconazole as second-line therapy for metastatic nonsquamous non-small-cell lung cancer. J. Thorac. Oncol. 8: 619–623. 10.1097/JTO.0b013e31828c3950 PMC3636564 23546045

[36] Kim J, Tang JY, Gong R, Kim J, Lee JJ, et al (2010) Itraconazole, a commonly used antifungal that inhibits Hedgehog pathway activity and cancer growth. Cancer Cell 17: 388–399. 20385363 10.1016/j.ccr.2010.02.027 PMC4039177

[37] Xu WS, Parmigiani RB, Marks PA (2007) Histone deacetylase inhibitors: molecular mechanisms of action. Oncogene 26: 5541–5552. 17694093 10.1038/sj.onc.1210620

[38] Landau DA, Carter SL, Stojanov P, McKenna A, Stevenson K, et al (2013) Evolution and impact of subclonal mutations in chronic lymphocytic leukemia. Cell 152: 714–726. 23415222 10.1016/j.cell.2013.01.019 PMC3575604

[39] Wang Y, Waters J, Leung ML, Unruh A, Roh W, et al (2014) Clonal evolution in breast cancer revealed by single nucleus genome sequencing. Nature Jul 30. doi: 10.1038/nature13600. [Epub ahead of print] 10.1038/nature13600 PMC4158312 25079324

[40] Berulava T, Ziehe M, Klein-Hitpass L, Mladenov E, Thomale J, et al (2013) FTO levels affect RNA modification and the transcriptome. Eur J Hum Genet. 21: 317–323. 10.1038/ejhg.2012.168 PMC3573201 22872099

[41] Reid MA, Wang WI, Rosales KR, Welliver MX, Pan M, et al (2013) The B55α subunit of PP2A drives a p53-dependent metabolic adaptation to glutamine deprivation. Mol Cell 50: 200–211. 23499005 10.1016/j.molcel.2013.02.008

[42] Singh B, Berry JA, Shoher A, Ayers GD, Wei C, et al (2007) COX-2 involvement in breast cancer metastasis to bone. Oncogene 26: 3789–3796. 17213821 10.1038/sj.onc.1210154

