# Supplementary material for: Highly Adaptable Triple-Negative Breast Cancer Cells as a Functional Model for Testing Anticancer Agents
Source: PLoS One. 2014 Oct 3;9(10):e109487. doi: 10.1371/journal.pone.0109487 (PMC4184880; doi:10.1371/journal.pone.0109487)
Supplement: Table S3 — Molecular Alterations Affecting Several Networks in MA2 cells, Related to Figure 2 . The significant alterations in gene expression in MA2 cells, which are listed in Table S2, were subjected to core analysis in the Ingenuity Pathway Analysis software. Significantly up-regulated or down-regulated molecules are grouped according to the diseases and functions they may impact. The analysis is composed of 25 networks. (PDF) [file pone.0109487.s003.pdf]

☐	△ ID	Molecules in Network	Score	Focus Molecul	Top Diseases and Functions
☑	1	↑ARID5B*, ↑BRWD1, ↑C15orf57, ↓CCND1, ↓CDCA2, ↑CDK20*, ↓CENPK, ↓CENPN, ↓CTNNAL1*, ↓DEPDC1, ↓DONSON, ↓EIF5A2, ↓ENDOD1, ↓ERI1 (includes EG:361159)*, ↑GPC6 (includes EG:10082)*, ↑KLHDC1, ↑KLHL24, ↓KRT80, ↑LCORL, ↑LIMCH1*, ↑LINC00467*, ↑LOC729678, ↑MBD5, ↑MESDC2, ↑PTOV1, ↑RBMS3, ↓RNF219, ↓RUFY3, ↓ST6GALNAC5, ↑TIGD1, ↑TMCC2, ↑TMEM219, ↓URGCP, ↓ZNF367, ↑ZNF783	31	35	Lipid Metabolism, Molecular Transport, Small Molecule Biochemistry
☐	2	↑ALS2CR8, ↑AZI2*, ↑BCKDHA, ↓CDKN2A, ↑CLN3 (includes EG:1201), ↑CUEDC1, ↑DECR2, ↑EML6, ↓FAM111A, ↓GPRC5A, ↑HS6ST3, ↓IGF2BP3, ↓INPP5E, ↓KCNK1, ↑LOC728392, ↑MAGED1, ↑N4BP1, ↓NEDD4L*, ↑NOVA1*, ↓NUP205, ↓ODZ2*, ↓ODZ3, ↓PHLDA3, ↓PMEPA1*, ↑PRR16, ↓PRRG1, ↓RAP1GDS1*, ↑RASL10B, ↓RASL11B, ↓RREB1, ↑SSR3, ↓VRK1, ↑ZDHHC1, ↓ZNF202, ↓ZNF526	31	35	Embryonic Development, Organ Development, Organ Morphology
☐	3	↓ANKRD13B, ↑APBB2, ↓ATP13A2, ↑BICC1, ↑CEND1, ↑CMTM6, ↑DOK6, ↓EGFR, ↑FBN2 (includes EG:14119), ↑GPM6B*, ↓KCTD9*, ↓LRIG1, ↑MATN2, ↓MATN3, ↓MCOLN2, ↓MCOLN3*, ↓MEGF6, ↓MFAP2, ↓MFAP5, ↑MT1M, ↑PCDHGA12, phosphoinositide phospholipase C, ↑PLCB4*, ↑PLCD4, ↓PLCH1, ↓ROS1, ↓SH2D3A, ↑SH3BGRL, ↓SH3D21*, ↑TMEM55A, ↑TMEM55B, ↑UROD, ↓VAC14, ↑YPEL5*, ↑ZFYVE28	28	34	Carbohydrate Metabolism, Lipid Metabolism, Small Molecule Biochemistry
☐	4	↑ARMCX5-GPRASP2/GPRASP2, ↑BAI2*, ↓CELSR2, ↓EMR2, ↑FPR3, ↑GLP2R*, Gpcr, ↑GPR4, ↓GPR17, ↑GPR20, ↓GPR21, ↑GPR64*, ↓GPR68, ↓GPR84, ↓GPR87, ↑GPR97, ↓GPR110, ↓GPR115, ↓GPR125, ↓GPR126, ↑GPR155*, ↓GPR160, ↓GPR161*, ↑GPR171, ↑GPR173, ↓GPR179, ↑GPR137B*, ↓GPRC5C, ↓LGR4, ↓LPAR3, ↓NTSR1, ↓OPN1MW/OPN1MW2, ↓QRFPR, ↓RXFP3, ↓TAS1R3	28	34	Cellular Movement, Carbohydrate Metabolism, Cell-To-Cell Signaling and Interaction
☐	5	↑AMOT, ↓AMOTL1, Cebp, ↓CRB3, ↑CYFIP2*, ↑DCAF7, ↑DENND3*, ↓DMBT1*, ↓DOCK7*, ↓IL8*, ↓INADL, ↓KIF13B*, ↓LLGL2*, ↑LONP2, ↑LURAP1, ↓MARK2*, ↑MARK4, ↓MYO18A*, ↓PARD6A, ↓PARD6B, ↓PHLPP1, ↓PHLPP2, ↓PRICKLE1, ↓SLC9A3R2, ↓SOGA2, ↑STRADA*, ↑STRADB, ↑TCP10/TCP10L2, ↓TEAD4, ↑TLE6*, ↑WBP1, ↓WWTR1, ↓YAP1 (includes EG:10413), ↑ZNF275, ↑ZNF821	28	34	Cardiovascular System Development and Function, Cell Death and Survival, Cell Morphology
☐	6	↓AADAT, ↑BEX2, ↑CNTN2, ↓CNTNAP2, ↑DMRTA2, ↑DRGX, ↓EPB41L2, ↑ETV1*, ↓F2RL1, ↑FAM194A, ↑FGF13, ↑HOXD1, ↑IRX3, ↓ISL1, ↑LDB1, ↓LHX1, ↑LHX2, ↓LILRB3*, ↑LMO1, ↓MSC, ↑MSI2, ↓PDLIM5*, ↓POU4F1*, ↓PPP1R1C, ↑PYGO1*, ↓RBM47*, ↑SKOR1, ↑SLC16A12, ↓SLC22A10, ↑SLC26A1, SOX2-OCT4-NANOG, ↑SSBP2, ↓VSTM2A, ↑ZKSCAN1, ↑ZNF354A	28	34	Nervous System Development and Function, Tissue Development, Embryonic Development
☐	7	↑ALPL, ↑BEX5, ↑BHLHB9, ↓BRI3BP, ↓C15orf48, ↓CDYL, ↓CPA4, ↑CPD, ↑CRISPLD2, ↑CYB5R2, ↓DNMT3B, ↑EPDR1, ↓ESRP2, ↓EZH2, ↑FBXO15, ↓FHOD3, ↑FOXD3, ↑GLT8D2, ↑HCP5, ↑ITFG1, ↑KCNQ1OT1, ↓LACC1, ↓LCN2, ↓LXN, ↑MBD4, ↑NAP1L2, ↑OXTR, ↓PCDH20, ↑PJA1, ↑PRAME*, ↑RAB39B, ↑SFRP2, SOX2-OCT4, ↓TAF1D, ↓ZNF692	28	34	Cancer, Gene Expression, Developmental Disorder
☐	8	↓ABTB2, ↑ANKRD42*, ↓BCL7A, beta-galactoside alpha-2,3-sialyltransferase, ↓C11orf82, ↑CD163L1, ↑CHI3L2, ↑CIB2, ↑CNPY3, ↑COLQ, ↑FBXO38, ↓FUT3, ↑HLA-J, ↓KLF7, ↓KMO*, ↑LOC285141, ↓LRRC8D, ↓MTHFD2L*, ↑NINJ1, ↑RAB37*, ↓RASSF7, ↓RHBDF2, ↓RTP3, ↑SCAMP2, ↓SLC16A5, ↑SLC43A2, ↑SLU7 (includes EG:10569), St3gal, ↑ST3GAL1, ↑ST3GAL3*, ↓ST3GAL4, ↓TNF, ↑VASH1, ↑WLS, ↓ZDHHC8	26	33	Post-Translational Modification, Cellular Movement, Hematological System Development and Function
☐	9	↓ADRA1B, ↑ARC, ↑C14orf1, ↑CCDC136*, ↑CRADD, ↑CRELD1, ↑DDAH1*, ↓DENR, ↑DISC1*, DNA-directed DNA polymerase, ↓DST*, ↑ETV3, ↑FAM190B, ↓GNPTAB*, ↓HYLS1, ↑KIF5A*, ↑KLHL20, Lamin, ↑MACROD2, ↑NEFL, ↓PAPD5, ↓PAPD7, ↓POLA2, ↓POLD1, ↓POLD3, ↓POLE2, ↓PRIM2, ↓SNX22, ↑SYNE1, ↑TDRD6, ↑TENC1, ↑TERF2, ↓TRIM47, ↑ZCCHC7, ↓ZNF365*	26	33	Developmental Disorder, Hereditary Disorder, Metabolic Disease
☐	10	↓B3GALNT1, Calbindin, ↓CTSL2, ↑HCFC2, ↑HCST, ↑HELZ, ↓HOXA9, ↓HOXA10, ↓HOXA11, ↑MEIS1, ↓MFNG, ↓MICB, ↓MLL*, ↓MYB*, ↓NBPF15 (includes others)*, ↑NDRG2*, ↑PBX1*, ↑PBXIP1*, ↓PCDH1, ↓PGBD3, ↑PHF20, ↑PHF20L1, ↓PLEKHF1, ↓RAD51AP1, ↑RCOR2, RNA polymerase II, ↓RNGTT, ↑SDHC*, ↓SERINC2, ↑ST13*, ↓STK17B*, ↓THOC1, ↓TMSB10/TMSB4X*, ↓TRHR, ↑TRIB2*	26	33	Cancer, Gene Expression, Cellular Growth and Proliferation
☐	11	↓AHR, ↑AOX1*, ↑ARNT2*, ↑ATOH8, ↑BCL3, ↑BCL6, ↑C11orf86*, ↑CD36, ↓COL13A1, ↑COL18A1, ↓COL27A1, ↑CYBASC3, ↑DACT2, ↑ECH1, ↑ENHO, ↑EPB41L5, ↓FBP1, HNF4α dimer, Hsp27, ↑IL6R, ↑LYST, ↑MFSD2A, ↑MLYCD, ↑NPAS1, ↑PDK4, ↑PMFBP1, ↓PPARD, ↑SDSL, ↑SPOCK1, ↑ST8SIA5, ↓THBS1*, ↑TMEM120A, ↓TNFAIP8L1, ↑ZBTB6, ↓ZDHHC14	26	33	Cellular Function and Maintenance, Lipid Metabolism, Molecular Transport
☐	12	↑ACOT8, ↓BTNL2, Ces, ↑CES1*, ↑CISD1, ↑CROT, ↓CSF2, ↑CYP27A1, ↓ENOSF1, ↓ETS1, ↓FAM129A, ↓FAM171A1*, ↓FBXL19-AS1, ↑FITM1, ↓GATA3, Growth hormone, ↑IL16, ↑INSIG1, ↑LEPR*, ↑LEPROT, ↑NR4A2*, ↑NUDT7, ↑PCTP, ↑PNPLA7, ↑PPARA, ↓PSMC3IP, ↑RNF139, ↑SATB1, ↑SCAMP1*, ↑STAT5B, ↓TGS1, ↑THRB, ↓UBE2I, ↑ZMAT2, ↑ZNF25*	26	33	Lipid Metabolism, Small Molecule Biochemistry, Molecular Transport
☐	13	↓AFMID, ↑ARHGAP4, ↑C11orf70*, ↑C16orf53, ↓C20orf72, ↑CASC4, ↓CCL5, ↑CD14, ↑CLCN4*, ↑CSH1/CSH2, ↓DDB2*, ↓E2F1, ↑FAM117A, ↓FAM167A, ↑FAM213A, ↓FRMD6, ↓GINS1, ↓GSG2, Histone h3, Histone h4, ↑KLHDC10, ↓LARP1B*, ↑NIPAL2, ↑ONECUT1, ↓ONECUT3, ↑PRL, ↓SETDB2, ↓TERT, ↑TOX2, ↓VDR, ↓VKORC1, ↑VWA5A*, ↑YPEL3, ↓ZDHHC11*, ↑ZNF790	26	33	Cancer, Cardiovascular System Development and Function, Organismal Development
☐	14	↑AGBL2, ↓ANXA8L2 (includes others), ↓CEP55, ↑CHIC1*, ↓DCLRE1A, ↓DSC3*, ↓E2F8, ↑FAM125A, ↑FRMD4A, ↓GTSE1, ↓IER5, ↑IKBIP*, ↑JMY, ↑LRRC17, ↓MAGEB1, ↓MBNL2, ↓MPV17L, ↓MTMR11, ↓NRARP, ↑PQLC3*, ↓RAD54L, ↓RASSF3*, ↓RBM38, ↓RNF144B, ↑SCN3B, ↑SHISA5, ↑SLC37A3, ↑TEP1 (includes EG:100321823), ↑TMEM43, ↑TP53 (includes EG:22059), ↓TTLL5, ↓UBA6, ↓VPS37A, ↓VPS37B, ↑VPS37D	25	35	Cell Death and Survival, Embryonic Development, Cellular Function and Maintenance
☐	15	↑ADAMTS9, ↑AMIGO1, ↓AMIGO2, ↑AMIGO3*, ↑ANO3, ↓ARHGAP10, ↑ATP6AP2, ↑B3GNT9, ↓CARD14, CD14/TLR4/LY96, ↑CGREF1, ↑CST7, ↓ERAP2, ↑FUCA1, IL1/IL6/TNF, ↓INF2*, ↑JKAMP, ↓KDM2A, ↑LCA5, ↑LY96 (includes EG:17087), ↓MLKL, NFkB (complex), ↓PKN3, ↑PPP1R16B, ↑RAB3C*, ↓RELT, ↑RNF112, ↓RNF19B, ↑SLC22A4, ↓STK40, ↑TMOD2, ↑TRAPPC9, ↑TRIM6, ↓WDR34, ↓ZDHHC3	24	32	Hereditary Disorder, Ophthalmic Disease, Developmental Disorder
☐	16	↑ADC*, ↓AGAP3, ↓ATP2C1, ↑C1QL1, ↓CASZ1, ↓CERS1, ↑CERS4, ↑CERS6*, ↓COBLL1, ↓E4F1, ↑ELOVL3, ↑FAM50A, ↓FLRT2, ↑FOS, ↑GAMT, ↓HOOK1*, ↑IDS*, ↓IQCJ-SCHIP1, ↑KBTBD10, ↓MGAT4A, ↓MPZL2*, ↓MPZL3*, ↑MT1A, ↓MTMR2*, ↓NPTX1, ornithine decarboxylase, ↑PAOX*, ↓POU4F3, ↑RGMA, ↑SBF2, Sos, sphingosine N-acyltransferase, ↑TPP1 (includes EG:1200), ↑VSX1, ↑ZNF652	24	32	Lipid Metabolism, Molecular Transport, Small Molecule Biochemistry
☐	17	AKAP, ↓BRF2, ↓CCDC64B, ↓CLCN2, ↑CRBN, ↑DOC2A, ↓EXPH5, ↑FAM165B, KDELR, ↑KDELR2, ↑KDELR3, ↑ORMDL1, ↓OTUD4*, Pka, ↑PLIN5, ↑RAB13, ↑RAB24, ↓RAB27B, ↓RAB3D, ↑RAB40B, ↑RPH3AL, ↑SAR1A*, ↑SERINC3, ↓SLC16A1, ↑SMAP1, ↑SNPH, ↓SPTBN2, ↑SSR4, ↑SURF4*, ↓SYTL1*, ↓SYTL2, ↑TMBIM4*, ↑TMCO1, ↑UNC13B*, ↓UNC13D	24	32	Cellular Function and Maintenance, Molecular Transport, Cellular Assembly and Organization
☐	18	↑AASS, ↑ARFGAP3, ↓ASAP2, COP I, ↑COPA, ↑COPG1, ↑COPG2, ↑COPZ2, ↑CREB3L2*, CRK/CRKL, ↓ENAH, ↓FAT1, ↓HES4, ↑KIF5C, ↓KLC3, ↑MIA3, ↓NUP85 (includes EG:287830), ↓PRRG2, ↓PXN*, ↑REPS2*, ↓SAPCD2, ↑SAR1B, ↑SEC13*, Sec23, ↑SEC16B, ↑SEC23A, ↑SEC23IP, ↑SEC24A*, ↑SEC24D, ↓TES, ↑TMED3, ↑TMED9, ↑TMED10*, ↑TMOD1, ↑TPPP	24	32	Cell Morphology, Cellular Assembly and Organization, Cellular Function and Maintenance
☐	19	↓ATAD3A/ATAD3B*, ↓CD52, ↓CDSN, ↑CHCHD6, ↑CHCHD3 (includes EG:296966), ↑CKB, ↓DYSF, ↑ETF1, ↑F2R*, ↑GNAI1, ↑GNG3, ↓GNG4, ↑GNG11, ↓LMNB2, ↑NUCB1, ↓ODF2L, Plc beta, ↓PLCB2, ↓PRSS2, ↓PRSS3, ↓PRSS23, ↓RECQL4, Serine Protease, ↑SERPINA3, ↓SLPI, ↓SMG1*, ↑SMG8, ↓SPINK1, ↓SPINK5, ↑STOM, ↓TELO2, ↑TMEM50B, Trypsinogen, ↓TTI2 (includes EG:100334230), ↑WBP5	24	32	Endocrine System Disorders, Gastrointestinal Disease, Hereditary Disorder
☐	20	Ant, ↑ARHGEF25*, ↑BOD1, ↓CCDC64*, Cytoplasmic Dynein, ↑DCTN4, ↑DYNC1I1, ↑DYNLRB2, ↑DYNLT3, ↓EHF, ↑EIF3K, ↓ELF3, ↓FAM83D, ↓FKBP4, ↓FLNB, ↑FOLH1, ↓KIF2C, ↑MAPRE3, ↑MTUS2, ↑NPHP1, ↓PAK1, ↓POU2F3, ↑RAB6B, ↑RPGRIP1L, ↑SIRT4, ↑SLC25A6*, ↓SLC25A10, ↓SPG7, ↓SPRR2A*, ↓TARS, threonine-tRNA ligase, ↓TIMM8B*, ↑TOMM20 (includes EG:100043869), ↓TROAP*, ↑ZNF655	24	32	Developmental Disorder, Hereditary Disorder, Neurological Disease
☐	21	↓ASPM*, ↓AURKB, ↑BTBD9, ↑CALCOCO1, ↓CASC5*, ↓CAV2, Caveolin, ↓CDCA8, ↓CEP78, DRD1/5, ↓GFM1, ↓GNAQ, ↑IFT122, ↑IFT140, ↓INCENP, ↓IQGAP3, ↑KLHL9, ↓KNTC1, ↓MAD2L1, ↓MALL, ↓NDC80, ↓NSUN2, protein-synthesizing GTpase, ↓PTRF, ↑RCN1 (includes EG:19672), ↑RSL1D1, ↑SCFD2, ↓SGOL1, ↓TACC3, ↓TRIM17*, ↑TTBK2, ↑TUB, ↑WDR19*, ↓ZW10, ↓ZWINT*	24	32	Cell Cycle, Cellular Assembly and Organization, DNA Replication, Recombination, and Repair
☐	22	ADRB, ↑AZGP1, ↓CALML5, ↓CASP14, ↑CORO2A*, ↑DZIP3*, G-protein beta, ↑HIST2H2AA3/HIST2H2AA4, ↑HIST2H2BE (includes others), ↓HR, ↓MID1 (includes EG:100330952), ↓MKRN3, ↑PARP3, ↓RFWD3, ↑RNF25, ↑RNF43, ↓RNF122, ↓RNF125, ↑RNF130*, ↑RNF150, ↓RNF165, ↓RNF216, ↑RNF144A, ↑TBL1X*, ↓TCN1, UBE2, ↑UBE2E2, ↓UBE2E3, ↑UBE2J1, ↑UBE2L6, ↑UBE2Z, ↑UFL1, ↓USP53*, ↓WFDC2*, ↓ZNRF1	24	32	Post-Translational Modification, Nutritional Disease, Dermatological Diseases and Conditions
☐	23	↓AFF1*, ↑BRD8, Caspase 3/7, ↑CCBE1, ↑CUL4B*, ↑DCAF5, ↑DCAF8, ↓DCPS, ↓DOT1L*, ↓DTL, ↑EAF1 (includes EG:306261), ↓EED, ↑GRASP, ↓GTPBP3, ↑HEXIM1, ↑HEXIM2, histone-lysine N-methyltransferase, ↓L3MBTL1, ↓LARP7, ↑MLLT6, ↓NARG2, ↑NSD1, P-TEFb, ↑PPP1R10, ↑S1PR3, ↓SETD8*, ↓SIAH2, ↑SUV420H1, ↓TLE1*, ↑TLE2, ↑TOX4, ↑TRIM9, ↓WDHD1, ↓WHSC1*, ↓WHSC1L1	24	32	Embryonic Development, Tissue Development, Cell Cycle
☐	24	↑ABHD6, ↓AMBRA1, ↓ASXL2, ↑BECN1, ↓CBX2, ↑CBX6, ↑CBX7, DUB, ELMO, ↑ELMO2, ↓ELMO3, ↓GIPC1, ↑HOXB6, ↑HOXD8, ↑HOXD9, ↓KIAA0226, ↓KIAA0226L, ↓MYO6, ↑NRBF2, ↑PCGF2, ↓PGAM5, ↑PHC1*, ↑PHF1, ↓PLEKHG6, ↑SPATA24, ↓SSX2IP, ↓TPBG, Trk Receptor, ↑UCHL1, ↓UCHL3, ↑USP11*, ↓USP28*, ↑USP30, ↑USP27X, ↑UVRAG	24	32	Cancer, Skeletal and Muscular Disorders, Tissue Morphology
☐	25	3 BETA HSD, ↓BCL11B*, Bcl9-Cbp/p300-Ctnnb1-Lef/Tcf, ↑BMF, ↓C10orf116, ↑CYP26B1, ↓EMP1, Endothelin, ↑FBLN5, ↑FOXL2, ↓FOXP1, ↑GHRL, ↑H1F0, ↑HBP1, ↑HOPX, ↑LTBP1*, ↓MIA, ↑NKX3-2, ↑NNMT, ↑OSR1, ↓OSR2, ↓PHEX, ↑PTPRN2, ↑RAD51B, ↑RAD51C, ↓SLCO4A1, ↑SND1, ↓SOX4, ↓SOX9, ↑SWSAP1, ↓TCF4, ↑TMEM37, ↓VNN1, ↓XRCC2, ↓XRCC3*	24	32	DNA Replication, Recombination, and Repair, Ophthalmic Disease, Digestive System Development and Function

©2000-2014 Ingenuity Systems, Inc. All rights reserved.
