# Supplementary material for: Highly Adaptable Triple-Negative Breast Cancer Cells as a Functional Model for Testing Anticancer Agents
Source: PLoS One. 2014 Oct 3;9(10):e109487. doi: 10.1371/journal.pone.0109487 (PMC4184880; doi:10.1371/journal.pone.0109487)
Supplement: Table S6 — Chromosomal Gains and Losses Shared by MA1 and MA2 Cells, Related to Figure 3 . The 13 aberrations (5 amplifications and 8 deletions) detected under stringent settings in both MA1 cells and MA2 cells are listed. The list includes all the genes located in the affected chromosomal regions. We extracted this information manually in Microsoft Excel from Tables S4 and S5. (DOCX) [file pone.0109487.s006.docx]

| **Chromosome** | **Cytoband** | **Start** | **Stop** | **Gene Names** |
| --- | --- | --- | --- | --- |
| **Amplified Regions** | | | | |
| chr11 | p15.4 | 10126389 | 10162706 | SBF2 |
| chr13 | q12.3 | 29884445 | 29925611 | MTUS2 |
| chr16 | q11.2 | 46593548 | 46652806 | ANKRD26P1, SHCBP1 |
| chr16 | q12.1 | 48664327 | 50286923 | CBLN1, C16orf78, ZNF423, TMEM188, HEATR3, PAPD5 |
| chr16 | q12.2 | 53469745 | 53779762 | RBL2, AKTIP, RPGRIP1L, FTO |
| **Deleted Regions** | | | | |
| chr5 | p15.33 - p15.1 | 1329465 | 16045771 | CLPTM1L, SLC6A3, LPCAT1, SDHAP3, LOC728613, MRPL36, NDUFS6, IRX4, IRX2, C5orf38, IRX1, LOC340094, ADAMTS16, KIAA0947, FLJ33360, MED10, UBE2QL1, LOC255167, NSUN2, SRD5A1, PAPD7, ADCY2, C5orf49, FASTKD3, MTRR, SEMA5A, SNORD123, TAS2R1, LOC285692, FAM173B, CCT5, CMBL, MARCH6, ROPN1L, ANKRD33B, DAP, CTNND2, DNAH5, TRIO, FAM105A, FAM105B, ANKH, FBXL7 |
| chr5 | p14.3 - p13.1 | 22549775 | 39072167 | CDH12, PRDM9, CDH10, CDH9, CDH6, RNASEN, C5orf22, PDZD2, GOLPH3, MTMR12, ZFR, SUB1, NPR3, C5orf23, TARS, ADAMTS12, RXFP3, SLC45A2, AMACR, C1QTNF3, RAI14, TTC23L, RAD1, BRIX1, DNAJC21, AGXT2, PRLR, SPEF2, IL7R, CAPSL, UGT3A1, UGT3A2, LMBRD2, MIR580, SKP2, C5orf33, RANBP3L, SLC1A3, NIPBL, C5orf42, NUP155, WDR70, GDNF, EGFLAM, LIFR, OSMR, RICTOR |
| chr5 | p13.1 - p11 | 39115101 | 46115086 | FYB, C9, DAB2, PTGER4, TTC33, PRKAA1, RPL37, SNORD72, CARD6, C7, HEATR7B2, C6, PLCXD3, OXCT1, C5orf51, FBXO4, GHR, CCDC152, SEPP1, C5orf39, LOC153684, ZNF131, MGC42105, HMGCS1, CCL28, C5orf28, C5orf34, PAIP1, NNT, FGF10, MRPS30, HCN1 |
| chr7 | p11.2 | 54258121 | 57621419 | HPVC1, VSTM2A, SEC61G, EGFR, LANCL2, VOPP1, LOC442308, FKBP9L, SEPT14, ZNF713, MRPS17, GBAS, PSPH, CCT6A, SNORA15, SUMF2, PHKG1, CHCHD2, LOC389493, LOC650226, DKFZp434L192, ZNF479, LOC642006, ZNF716 |
| chr11 | p15.4 | 2916747 | 2942557 | SLC22A18AS, SLC22A18 |
| chr11 | p15.4 - p15.2 | 10173030 | 13550146 | SBF2, ADM, AMPD3, RNF141, LYVE1, MRVI1, CTR9, EIF4G2, SNORD97, ZBED5, GALNTL4, CSNK2A1P, USP47, DKK3, MICAL2, MICALCL, PARVA, TEAD1, RASSF10, ARNTL, BTBD10, PTH |
| chr13 | q33.2 - q34 | 105177575 | 115083342 | DAOA, EFNB2, ARGLU1, FAM155A, LIG4, ABHD13, TNFSF13B, MYO16, IRS2, COL4A1, COL4A2, RAB20, CARKD, CARS2, ING1, C13orf29, ANKRD10, ARHGEF7, C13orf16, SOX1, C13orf28, TUBGCP3, C13orf35, ATP11A, MCF2L, F7, F10, PROZ, PCID2, CUL4A, LAMP1, GRTP1, ADPRHL1, DCUN1D2, TMCO3, TFDP1, ATP4B, GRK1, LOC100130386, FAM70B, GAS6, FLJ44054, RASA3, CDC16, UPF3A, ZNF828 |
| chr16 | p13.12 | 14377515 | 14760662 | MIR193B, MIR365-1, PARN, BFAR |

**Table S5. Chromosomal Gains and Losses Shared by MA1 and MA2 Cells, Related to Figure 3**
